# Long Time Scale Ensemble Methods in Molecular Dynamics: Ligand–Protein Interactions and Allostery in SARS-CoV-2 Targets

**DOI:** 10.1021/acs.jctc.3c00020

**Published:** 2023-05-29

**Authors:** Agastya P. Bhati, Art Hoti, Andrew Potterton, Mateusz K. Bieniek, Peter V. Coveney

**Affiliations:** † Centre for Computational Science, Department of Chemistry, University College London, London WC1H 0AJ, United Kingdom; ¶ Computational Science Laboratory, Institute for Informatics, Faculty of Science, University of Amsterdam, Amsterdam 1098 XH, The Netherlands; § Advanced Research Computing Centre, University College London, London WC1H 0AJ, United Kingdom

## Abstract

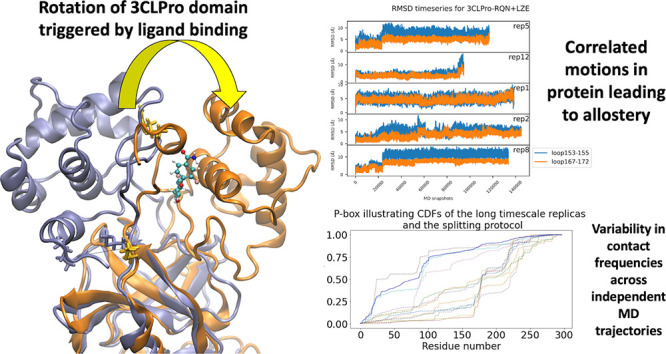

We subject a series of five protein–ligand systems which contain important SARS-CoV-2 targets, 3-chymotrypsin-like protease (3CLPro), papain-like protease, and adenosine ribose phosphatase, to long time scale and adaptive sampling molecular dynamics simulations. By performing ensembles of ten or twelve 10 μs simulations for each system, we accurately and reproducibly determine ligand binding sites, both crystallographically resolved and otherwise, thereby discovering binding sites that can be exploited for drug discovery. We also report robust, ensemble-based observation of conformational changes that occur at the main binding site of 3CLPro due to the presence of another ligand at an allosteric binding site explaining the underlying cascade of events responsible for its inhibitory effect. Using our simulations, we have discovered a novel allosteric mechanism of inhibition for a ligand known to bind only at the substrate binding site. Due to the chaotic nature of molecular dynamics trajectories, regardless of their temporal duration individual trajectories do not allow for accurate or reproducible elucidation of macroscopic expectation values. Unprecedentedly at this time scale, we compare the statistical distribution of protein–ligand contact frequencies for these ten/twelve 10 μs trajectories and find that over 90% of trajectories have significantly different contact frequency distributions. Furthermore, using a direct binding free energy calculation protocol, we determine the ligand binding free energies for each of the identified sites using long time scale simulations. The free energies differ by 0.77 to 7.26 kcal/mol across individual trajectories depending on the binding site and the system. We show that, although this is the standard way such quantities are currently reported at long time scale, individual simulations do not yield reliable free energies. Ensembles of independent trajectories are necessary to overcome the aleatoric uncertainty in order to obtain statistically meaningful and reproducible results. Finally, we compare the application of different free energy methods to these systems and discuss their advantages and disadvantages. Our findings here are generally applicable to all molecular dynamics based applications and not confined to the free energy methods used in this study.

## 1 Introduction

There is an urgent need for drugs which target SARS-CoV-2, the pathogen responsible for the current coronavirus pandemic. In this regard, a concerted global effort has led to a rapid rise in the number of SARS-CoV-2 protein structures available in the Protein Data Bank (PDB), rendering the virus increasingly susceptible to rational, structure-based drug discovery. The typical timeline for the development of a single drug is 10–15 years, with an associated cost of $2 billion.^1^ In the face of the global COVID-19 pandemic, it is clear that the average development time scale of up to 15 years is wholly inadequate. It is therefore of crucial humanitarian and societal importance to develop new *in silico* workflows that accelerate the rate and enhance the quality of lead drug molecule design. Workflows which tie both artificial intelligence (AI) and molecular dynamics (MD) based methods together are required as no single methodology can achieve both the required accuracy and speed.^2^ While AI based methods can rapidly sample significant regions of chemical space in a short time frame, MD based methods (which are significantly lower in throughput) are able to predict ligand binding free energies to much higher accuracy.^3^ Furthermore, MD based methods have the potential to elucidate ligand binding kinetics and processes. The information derived from these simulations can be used to inform drug molecule optimization for improved kinetic and thermodynamic binding properties. In turn, MD based methods form a crucial part of modern drug discovery workflows. In the present work, we investigate the application of molecular dynamics (MD) simulations to the robust and reproducible elucidation of ligand binding mechanisms, sites, and interactions.

Molecular dynamics methods which aim to simulate the spontaneous process of protein–ligand binding have been in development for the past decade.^4−11^ Over this period, significant advancements have been made due to increased access to high-performance computing resources (in particular GPU accelerated hardware), improvements in computational hardware,^12,13^ and developments in MD algorithms.^14^ Thus, far, work in the field has predominantly focused on determining the mechanism of ligand binding to crystallographically determined sites.^4,5,7−10^ The idea behind these efforts is that by observing the spontaneous process of binding to these sites, key metastable states and associated protein ligand contacts can be identified. It is hoped that these interactions can then be modulated to optimize the kinetic and thermodynamic properties of drug binding.^4,5,7−10^ Some of these studies have also led to the elucidation of nonexperimentally determined sites, which may act as allosteric sites^5,6^ for the modulation of protein activity.

A central problem that arises in these studies is that they utilize protocols which do not systematically account for the chaotic nature of molecular dynamics simulation.^15^ The extreme sensitivity of such simulations to their initial conditions causes the many one-off results reported to be inherently nonreproducible.^16,17^ Addressing this issue forms the central focus of this work. The question which we address is whether it is possible to develop reliable methods that can accurately and reproducibly identify the full range of binding sites and binding modes that are accessible to a ligand. Such a method will permit us to go beyond what is essentially anecdotal evidence, and to report findings that are statistically reliable and of scientific value. We would like to remind readers that it is common to work with fixed epistemic parameters in molecular dynamics. Although a full uncertainty quantification analysis would require one to investigate their role in determining the uncertainties in quantities of interest, we have previously shown that the aleatoric uncertainty in MD simulations typically overwhelms that from the epistemic sources and that the latter’s uncertainty is damped in the output quantities of interest.^18^ Therefore, our focus in this work is only on the aleatoric uncertainty.

In general, spontaneous ligand binding methods work by initiating a molecular dynamics protein–ligand system from a configuration where the ligand is placed at some distance from the surface of the protein. During the simulation, the ligand explores the surface of the protein and binds to potentially druggable sites which may be orthosteric, allosteric, or even cryptic in nature.^6^ By analyzing the trajectories using methods such as Markov state model (MSM) analysis,^19,20^ thermodynamic and kinetic observables which are of key importance to the process of drug discovery can be extracted from the data. These include binding free energies,^21^ dissociation constants (*K*_d_)^21^ and on and off rates of binding (*K*_on_ and *K*_off_).^22,23^

When conducting these studies, the question arises as to whether the trajectory has sufficiently sampled phase space such that the probability distribution of the trajectory has converged to the equilibrium probability distribution of the protein–ligand system. Only this distribution would allow the true expectation values of the observables to be obtained.^15^ To sample the phase space, one of two distinct approaches is usually followed. In the first, which we term the “long time scale” regime, authors report several microsecond time scale simulations^5−7,21,24^ and, from these, compute the observables of interest. These observables include 3-dimensional ligand occupancy maps, ligand binding free energies, along with ligand association mechanisms and pathways. In the second regime, termed “adaptive sampling”,^9−11,23,25,26^ many simulations of shorter time scale are executed, and new simulations are adaptively initiated from specific simulation snapshots in order to “more thoroughly” explore regions of phase space that are of interest. Incidentally, many studies from the second regime report aggregate simulation times that fall in the microsecond time scale; this is misleading as performing a single simulation of that duration is not the same as we will discuss in detail in the current study. We note that some studies also combine the two techniques, using adaptive sampling to initiate new, “short” simulations from long time scale simulations that are stuck within nonproductive kinetic traps.^22^ Generally, this approach is taken in order to converge transition probabilities between metastable states that are identified during Markov state modeling.^27,28^

We would like to point out here that there are several accelerated sampling protocols that involve performing “ensembles” of simulations. These include methods that do not employ any external force or heating and just enhance sampling by performing multiple independent MD simulations concurrently with different starting conditions. Examples include ensemble dynamics,^29−35^ Markov state model (MSM),^19,20,27,28,33,35−38^ weighted ensemble (WE),^39−48^ and multilevel splitting (MS)^49−53^ methods. Although these methods involve performing “replicas” and generating “ensembles”, the fundamental question is whether we get the same answer (within error bars) on repeating the entire protocol using one of the above methods. Given that the dynamics is chaotic, it is expected that this is not the case and ensembles must be used as each execution of such a protocol would have a different initial condition.^15,18^ One example is replica exchange methods^54,55^ that also involve performing multiple MD simulations in parallel (so-called “replicas”). We have shown in previous work that on repeating a replica exchange calculation multiple times, we indeed observe variation in the outcome, and hence it is necessary to perform ensembles of the entire protocol (which itself contains “replicas”) to perform a systematic uncertainty quantification (UQ).^56^ Similar studies are required for other methods involving “ensembles” in order to properly assess UQ in those cases.

The purpose of the present paper is to systematically assess the distribution of properties obtained in long time scale simulations. By performing ensembles of ten to twelve 10 μs unbiased simulations, we are able to evaluate the utility of running individual long time scale simulations, investigate their reproducibility, and compare the results obtained from them to an “adaptive sampling” scheme which consists of 9 μs of *aggregate* simulation time. This is of interest as the wall time required to execute the long time scale runs is significantly longer than the wall time required for the entire adaptive sampling protocol (differences are on the order of weeks).

In our study, we apply these statistically robust techniques to three crucial SARS-CoV-2 drug targets: adenosine ribose phosphatase (ADRP),^57^ papain-like protease (PLpro)^58^ and 3-chymotrypsin-like protease (3CLpro).^59^ Each of these are globular, nonstructural proteins encoded by SARS-CoV-2 which play key roles in the lifecycle of the virus and serve as important potential targets for SARS-CoV-2. Our findings here shed light on potentially druggable sites on the surface of the proteins, elucidate relative binding free energies between each of the sites, and demonstrate binding mechanisms which may explicitly inform future efforts in SARS-CoV-2 drug discovery. We also compare different free energy protocols and discuss the applicability of each in different scenarios. In addition to these methodological developments, we report new scientific findings on the allosteric effects observed in 3CLPro. We discover conformational changes occurring at the active site of 3CLPro caused by the binding of a ligand at an experimentally known allosteric binding site and establish its relation to the inhibitory effect of that ligand. We demonstrate how these changes affect the binding of ligands at the main (active) site by distorting the binding pose. In addition, we also discovered a novel allosteric mechanism of action for a ligand that is hitherto known to act only by binding at the substrate binding site and blocking the catalytic dyad.

## 2 Theory

The present study approaches the subject of spontaneous protein–ligand binding simulations from the perspective of chaos theory and uncertainty quantification. In this section, we describe how the chaotic nature of molecular dynamics simulations causes individual simulations to be nonreproducible, no matter their length. We also describe how running ensembles of simulations remedies this by allowing for expectation values to be subjected to rigorous uncertainty quantification and convergence analysis. By presenting this theory, we make clear that running an ensemble of simulations which sum to a certain time is not equivalent to simply running a single simulation of the same aggregate time. The novel point which we explicitly demonstrate is that, contrary to the current consensus, the level of certainty of a simulation derived expectation value does not increase with simulation time. This necessitates the use of ensembles when reporting macroscopic expectation values for all long time scale simulations. Not only is such a prescription required by the tenets of statistical mechanics, it is also essential in order to quantify the uncertainty of the calculated properties.

### 2.1 MD Simulation and Equilibrium

In statistical mechanics, the value of an observable (*G*) of a dynamical system is derived by calculating the expectation value of the observable ⟨*G*⟩_*t*_ over the trajectory that the dynamical system takes through phase space
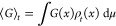
1The ergodic theorem, often used to justify the accuracy of “long time scale” molecular dynamics simulations, states that in the long time limit, the time average of a dynamical observable will approach its ensemble average. Namely,
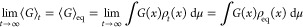
2where ρ_*t*_ and ρ_eq_ are respectively the (6*N* + 1)-dimensional time dependent and 6*N*-dimensional equilibrium probability distributions of the dynamical system. This implies that ρ_*t*_ has asymptotically approached ρ_eq_ (where the evolution of ρ_*t*_ is determined by the Liouville equation^15^).

Problematically for those working in the field of MD simulation, this assumption only holds true for time scales that are on the order of a Poincaré recurrence time, which is longer than the age of the universe.^60^ Therefore, because for any realistically obtainable single trajectory of a dynamical system ρ_*t*_ does not asymptotically approach ρ_eq_, the value of a given observable obtained from an individual molecular dynamics trajectory cannot be equated to the true value of the observable that would arise if phase space were ergodically sampled. Furthermore, the equality also requires that the dynamical system is mixing. In the ergodic hierarchy, mixing is a stronger property than ergodicity and is dependent on the system being chaotic.^15^

### 2.2 Uncertainty Quantification

Uncertainty quantification (UQ) is a field of endeavor that aims to analyze the interplay between simulation inputs and outputs for the purpose of determining the uncertainty associated with obtained results.^60^ In the present study, we are particularly interested in quantifying the aleatoric output uncertainty that is controlled by the initial random velocity seed. A series of our studies have presented robust evidence that simulation outcomes are strongly controlled by the initial random seed, and that averaging over a set of simulations all starting with different random seeds consistently reduces the uncertainty of obtained results.^61^ Indeed, this aleatoric uncertainty completely dominates the epistemic uncertainty arising from the way in which the model is parametrized and set up.^18^ A crucial feature of performing ensembles of simulations, which allows us to conduct uncertainty quantification, is that the *distribution* of properties of interest can be obtained. Here, we apply UQ to multiple properties of spontaneous ligand–protein binding simulations, namely, the protein–ligand residue contact frequency distribution and the computed binding free energy of the ligand with a protein target.

### 2.3 SARS-CoV-2 Protein–Ligand Systems

Three important SARS-CoV-2 targets form the focus of this work: 3CLpro, PLpro, and ADRP. 3-Chymotrypsin-like protease (3CLpro, also known as the main protease or nonstructural protein 5 (nsp5)), and papain-like-protease (PLpro, the protease domain of nsp3) are both proteolytic enzymes of SARS-CoV-2 which are responsible for cleaving the viral polyprotein chain (encoded by SARS-CoV-2 RNA) into nonstructural proteins that are required for the process of viral replication.^58,59^ Adenosine ribose phosphatase (ADRP) is a domain of nsp3 that is capable of interfering with the host immune response by removing ADP-ribose from ADP-ribosylated proteins and RNA.^57^ Thus, each of these protein targets are of considerable interest for SARS-CoV-2 drug design.

In a recent study by our group,^62^ 14 compounds of interest, each of which bind to one of three sites (the substrate binding site, allosteric site I, and allosteric site II) on the surface of 3CLpro, were identified from a previously conducted high-throughput crystallographic screen of repurposed drug molecules.^63^ Based on the results derived in that study, we selected 3 ligands of interest, MUT056399 (RQN), AT7519 (LZE), and pelitinib (93J) for the current study covering all three binding sites and a wide range of EC_50_ values (Table 1). Furthermore, by building the system containing both RQN (which binds to the substrate binding site)^63^ and LZE (which binds to allosteric site 2),^63^ we aim to capture whether the binding of RQN is affected by the binding of LZE, and if so then determine the allosteric mechanism involved in the process. For the PLpro system, we decided to focus on the ligand GRL0617 as it showed strong antiviral activity using NMR data and a promising value of EC_50_.^58^ Tofacitinib, which is a FDA approved pharmaceutical that is used to treat rheumatoid arthritis and ulcerative colitis,^64,65^ was chosen as the ligand for the ADRP system.

**Table 1:** Protein Targets and Their Corresponding Ligands

target name	compound name	PDBe^a^	exp. binding site	PDB ID	EC_50_ (μM)
ADRP	tofacitinib	MI1	N/A	6W02^b^	N/A
PLPro	GRL-0617	TTT	USP	7CJM	21.00
3CLPro	MUT056399	RQN	SB	7AP6	38.24
	pelitinib	93J	AS I	7AXM	1.25
	AT7519	LZE	AS II	7AGA	25.16

a PDBe ligand codes.

b 6W02 is the structure of ADRP bound to ADP ribose, not to tofacitinib.

## 3 Methods

We use ensembles of replica simulations (which here differ only in their initial particle velocities, drawn randomly from a Maxwell–Boltzmann distribution) in order to converge the statistics of the observable of interest. While previous studies by our group have investigated the necessity of ensembles for accurate and precise ligand binding free energy calculations,^14,66^ here we aim to demonstrate that ensembles of MD simulations are equally essential for the accurate determination of ligand binding sites and ligand–protein interaction mechanisms. To do this, we conduct a thorough comparative analysis of two alternative ensemble protocols: the long time scale protocol and the splitting protocol (an adaptive sampling method). These protocols are applied with a key goal in mind: to elucidate novel ligand binding sites and mechanisms for the three aforementioned proteins that are essential to the life cycle of SARS-CoV-2: ADRP, 3CLPro, and PLPro. We also employ different free energy protocols in order to determine the pros and cons of each method and discuss their domains of applicability and limitations.

### 3.1 Protein–Ligand Systems

**Figure 1:**
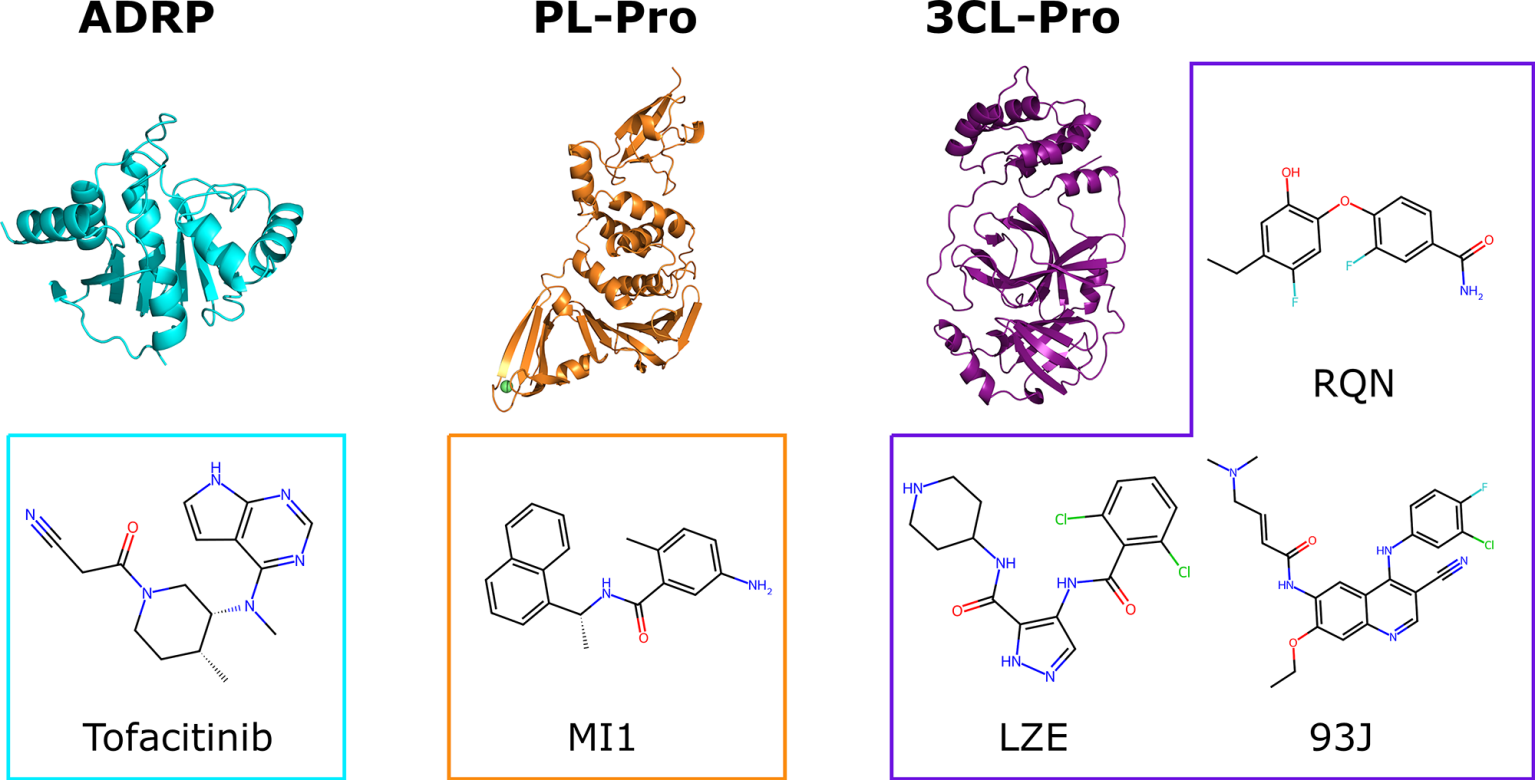
Structures of protein targets and corresponding ligands. ADRP (PDB ID: 6W02) is shown in cyan, PLpro (PDB ID: 7CJM) in orange, and 3CLpro (PDB IDs: 7AP6, 7AXM, 7AGA) in purple.

All three protein systems were selected due to their key-role in the life-cycle of the SARS-CoV-2 virus (as discussed in section 2). The protein structures were initially sourced from the PDB (see Table 1). All mutations in the initial crystallographic structures were back-mutated using the “Rotamers” tool in UCSF Chimera.^67,68^ Following this, ligands and other unwanted molecules were removed from the structures. The 3-dimensional conformers of the selected ligands were sourced from PubChem (https://pubchem.ncbi.nlm.nih.gov) and inserted into the system. Five protein–ligand systems were built in total. All systems are detailed in Table 1, and each of the proteins and ligands are shown visually in Figure 1. For each of our protocols and systems, the ligand was initially placed 20 Å away from the surface of the protein. A distance of 20 Å was chosen to minimize sampling bias that would arise from the initial position of the ligand due to long-range protein–ligand interactions. Thus, if the ligand was initially placed 3 Å from the binding site, it would immediately form interactions with the protein in that region and therefore most likely bind to that site. By distancing the ligand, we ensure that it stochastically diffuses around the protein before establishing its initial contact. Furthermore, the choice of separating the ligand and the protein by a distance of 20 Å is compatible with standard practice in the field, which is to distance the ligand between 20 and 30 Å away from the surface of the protein.^4,5,8,9^ Following this, each system was solvated using the TIP3P water model and charge-neutralized by inserting sodium or chloride ions.^69^

### 3.2 Simulations

In this subsection, we describe the two simulation approaches which we directly compare within this study: long time scale and splitting protocols.

#### 3.2.1 Long Time Scale Protocol

In the long time scale protocol, we perform ten or twelve replica simulations of 10 μs each. Each simulation is initiated from a common configuration in which the ligand is placed 20 Å from the surface of the protein with initial velocities drawn randomly from Maxwell–Boltzmann distribution. A simulation length of 10 μs is chosen on the basis that it is on the order of simulation times (microseconds to tens of microseconds) that have been utilized in multiple previous studies to derive information on the nature of ligand–protein interactions^6,7,21^ This protocol allows us to address two crucial aims within our study. First, we intend to identify whether a single 10 μs run can reliably reproduce the full range of binding sites and binding modes sampled by the aggregate of the “splitting” protocol (which has a length of 9 μs). And second, we aim to demonstrate the variability between the 10 μs members of the ensemble in order to examine whether a single “long time scale” (10 μs) trajectory is capable of generating reproducible and therefore reliable results. Indeed, as we show, each 10 μs run exhibits different statistics due to the chaotic nature of MD trajectories. A few recent papers from the D. E. Shaw group implicitly recognize such variability in MD simulations at the microsecond time scale.^70,71^ However, this has not been studied systematically hitherto, nor has the importance of ensembles of simulations at long time scale been discussed in the literature as we do in this study.

#### 3.2.2 Splitting Protocol

During the splitting protocol, 20 replica trajectories of 200 ns each are initiated from a common configuration in which the ligand is placed 20 Å from the surface of the protein. The initial particle velocities of each ensemble member are drawn randomly from a Maxwell–Boltzmann distribution. Trajectories are analyzed using RMSD heat maps, and the 5 replicas with the most kinetically stable poses in the final frame are chosen as configurations from which to initiate new sets of replicas, which we term “subreplicas”. We quantify “kinetic stability” by computing the ligand RMSD relative to the final frame of the simulation and select the five replicas which have an RMSD of <5 Å for the longest duration of time relative to the final frame. Each set of “subreplicas” contains 10 subreplicas of 100 ns each. The aggregate simulation time across the length of this protocol is 9 μs. Within the protocol, 200 and 100 ns were chosen as the simulation times as these are representative of the simulation time scale executed by those who have utilized ensemble based adaptive sampling protocols. Examples of this include the seminal study in the field by Buch et al. where 495 trajectories of 100 ns each were executed,^4^ among other papers which run on similar time scales.^8,9^ The purpose of the splitting method is to explore and identify as many binding sites as are feasible to which the ligand of interest may bind on the protein, while reducing the amount of wall time required to do so.

#### 3.2.3 Simulation Details

NAMD 2^69,72^ and OpenMM^73^ were used to run our simulations. All splitting protocol simulations were executed on Scafell Pike (hartree.stfc.ac.uk) whose compute nodes are comprised of Bullsequana X1000 (Intel Xeon processors and NVIDIA Tesla V100 accelerators). Long time scale runs for ADRP were also executed on Scafell Pike using OpenMM. All other long time scale simulations were executed using OpenMM on Summit (https://www.olcf.ornl.gov/summit) where compute nodes consist IBM Power9 processors and NVIDIA Tesla V100 accelerators. Force fields and modifiable simulation parameters were kept constant across MD engines and HPC platforms. All ligands (Table 1 and Figure 1) were parametrized in AmberTools using AM1-BCC charge assignments. The Amber FF14SB force field was used to parametrize the protein, and TIP3P water molecules were used to solvate the system. During equilibration, we conducted 1000 steps of energy minimization and then, in the NVT ensemble, applied harmonic constraints to protein and ligand atoms, while heating the system from 60 K to 310 K (an increase of 1 K every 2 ps). We then ran in the NVT ensemble at 310 K for 300 ps with no constraints. Following this, we performed equilibration in the NPT ensemble, using a Monte Carlo barostat with a pressure of 1.01325 bar and frequency of 50 fs. We reduced the strength of all harmonic constraints by half every 0.1 ps, 10 times. Subsequently, constraints were set to 0. Finally, the system was equilibrated without constraints at 310 K in the NPT ensemble for 1 ns. For all production and equilibration simulations, a Langevin thermostat was employed with a 2 fs time step together with a friction coefficient of 1/ps to simulate the dynamics of the system.

### 3.3 Ligand–Protein Contact Frequency Analysis

Ligand–protein residue contact frequencies are computed using a series of custom python scripts. The original scripts were written for the “getcontacts” tool by Dror et al.^74^ A contact between the ligand and the protein is defined as a van der Waals interaction, where the distance (|*AB*|) between two non-hydrogen atoms, *A* (belonging to the ligand) and *B* (belonging to the protein), satisfies the equation: |*AB*| < *R*_vdW_(*A*) + *R*_vdW_(*B*) + 0.5 (Å), where *R*_vdW_ is the van der Waals radius of the atom.

Upon computing the percentage of frames in which contacts are formed between the ligand and each protein residue for all of our trajectories, we obtain a two-dimensional matrix containing *m* × *n* elements where *m* is the number of trajectories executed and *n* is the number of residues in the protein. An element (*m*, *n*) of the matrix therefore corresponds to the contact frequency of the ligand with residue *n* in trajectory *m*. All ligand–residue contact frequency distributions are computed from these matrices using Python. These distributions provide meaningful and easily interpretable low dimensional representations of phase space sampling.

### 3.4 Binding Free Energy Calculations

To determine the relative binding free energy of a specific ligand for each of its identified binding sites, we use two protocols: ESMACS^66^ and the so-called “direct” binding free energy calculation method.^21,75^ By running the direct protocol, we also derive insights into the reproducibility of expectation values that are computed from “converged” simulations that are multiple microseconds in length.

#### 3.4.1 Enhanced Sampling of Molecular Dynamics with Approximation of Continuum Solvent (ESMACS)

Enhanced sampling of molecular dynamics with approximation of continuum solvent (ESMACS) calculations are fundamentally based on the Molecular Mechanics Poisson–Boltzmann/Generalized Born Surface Area (MMPB/GBSA) binding free energy calculation method.^76^ MMPB/GBSA calculations were conducted using AmberTools 20.^77^ For all MMPB/GBSA calculations, the 1-traj protocol was used, allowing the MMPB/GBSA ligand binding free energy (Δ*G*_MMPB/GBSA_) to be calculated from a single trajectory of the protein–ligand complex. Within the 1-traj protocol, Δ*G*_MMPB/GBSA_ is computed using the equation
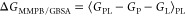
3where *G*_PL_, *G*_P_, and *G*_L_ correspond to the free energy contributions of the complex, protein, and ligand, respectively. Angular brackets denote that Δ*G*_MMPB/GBSA_ is computed as the average over all input snapshots, while the subscript “PL” denotes that the snapshots are taken from a single simulation of the protein–ligand complex. *G*_PL_, *G*_P_, and *G*_L_ are calculated using the following equation:
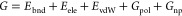
4where *E*_bnd_, *E*_ele_, and *E*_vdW_ are the bonded, electrostatic, and van der Waals terms, respectively. *G*_pol_ is the polar solvation free energy, and *G*_np_ is the nonpolar solvation free energy.

For each binding site identified during our long time scale and splitting protocols, we ran an ensemble of 25 4 ns trajectories. Since the predominant ligand binding sites and poses were identified as the final frames from which subreplicas were initiated in the splitting protocol, we used these configurations as the starting structure for ESMACS calculations performed for the ADRP system.

Our choice of running 25 simulations of 4 ns each is in accordance with previous findings by our group showing that 25 replicas of 4 ns are sufficient to obtain converged values of Δ*G*_ESMACS_.^66^ These trajectories were postprocessed in MMPBSA.py to produce 25 binding free energy estimates, one for each replica within the ensemble. The reported Δ*G*_ESMACS_ is the mean of the sampling distribution of means for this sample of 25 free energy estimated obtained using bootstrapping. The associated error bars are the corresponding standard errors.

#### 3.4.2 “Direct” Binding Free Energy Calculations

The “direct” binding free energy calculation method was originally developed by De Jong et al.^75^ and later applied to 10 μs trajectories by Pan et al. in 2017.^21^ We would like to point out that the method is justified on the basis that a sufficiently long single trajectory can be averaged to produce a meaningful macroscopic free energy. However, we will demonstrate that this assumption is not valid, and hence free energies computed through this method using a single trajectory are not reliable. To calculate the binding free energy, we use the following equations which were derived via statistical mechanics by De Jong et al.:^75^
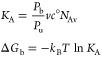
5Here, *P*_b_ and *P*_u_ are the fraction of simulation time in which the ligand is bound and unbound to the binding site of interest, respectively, *v* is the volume of the simulation box (L), *c*° is the standard-state concentration (1 mol L^–1^), *N*_Av_ is Avogadro’s number, *k*_B_ is Boltzmann’s constant, and *T* is the temperature (K). We define the ligand to be in the bound state when the first two closest distances between the heavy atoms of the ligand and the side chain heavy atoms of the binding site residues are <5 Å. All other frames are defined as unbound.

### 3.5 The Kolmogorov–Smirnov Test

To compare the ligand–residue contact frequency distributions, we perform the pairwise Kolmogorov–Smirnov (KS) test. The test compares the underlying continuous distributions *F*(*x*) and *G*(*x*) of two independent samples (in this case, two ligand–residue contact frequency distributions, each derived from separate MD trajectories and computed as described in section 3.3). Since the test is nonparametric, it is particularly suited to the comparison of ligand–residue contact frequency distributions as they have multiple peaks and are not normally distributed. To test the statistical certainty of two distributions being different from one another, we use the two sided *p*-test. For this test, the null hypothesis is that both of the distributions are sampled from the same underlying distribution. All KS tests are computed using the SciPy package in Python.^78^

## 4 Results and Discussion

This section is divided into two subsections. In the first subsection, we discuss aspects of our results that are important from the point of view of developing new scientific methods that yield statistically robust and reliable outcomes. We report our findings on the effect of stochasticity in MD simulations at “long” time scales. We show how this intrinsic characteristic of MD can be used to our advantage in order to enhance the sampling of phase space through introduction of biases. Further, we determine binding affinities using two different methods and compare them to discuss the advantages and disadvantages of each method and highlight scenarios where a particular method should be preferred. In the second subsection, we discuss the important scientific findings of our study. We discuss the novel allosteric mechanisms uncovered using our simulations that help us understand the inhibitory effects of RQN and LZE. We would also like to highlight here that, although the ADRP–tofacitinib complex has not been reported experimentally, it has still been included in this study as our main focus is on methodological advances and our findings in this regard hold true irrespective.

### 4.1 Development of Scientific Methods

#### 4.1.1 Aleatoric Uncertainty in “Long” MD Simulations

We have shown that classical molecular dynamics simulations are extremely sensitive to their initial conditions given their chaotic nature due to which two independent MD trajectories diverge exponentially with time.^15^ This has been exhibited in several published studies for short simulations (up to a few nanoseconds) including ours.^16,61^ Unprecedentedly, in this study we provide evidence for such divergence between independent simulations extending up to 10 μs. Our results conclusively show that MD trajectories lead to very distinct regions of a given phase space even when they are considered “long”. Thus, results based on one-off “long” simulations are at least as unreliable as one-off “short” simulations. Indeed, it is essential to perform ensembles in all cases to quantify the uncertainty and ensure reproducibility of results. This is due to the mixing nature of the dynamics which is a necessary and sufficient condition to reach equilibrium.^15,60^ We would like to note here that the extent of uncertainty (and hence the appropriate size of ensembles) depends on the free energy landscape. For small and/or rigid systems, uncertainties would be much smaller as compared to large and complex protein–ligand systems.

**Figure 2:**
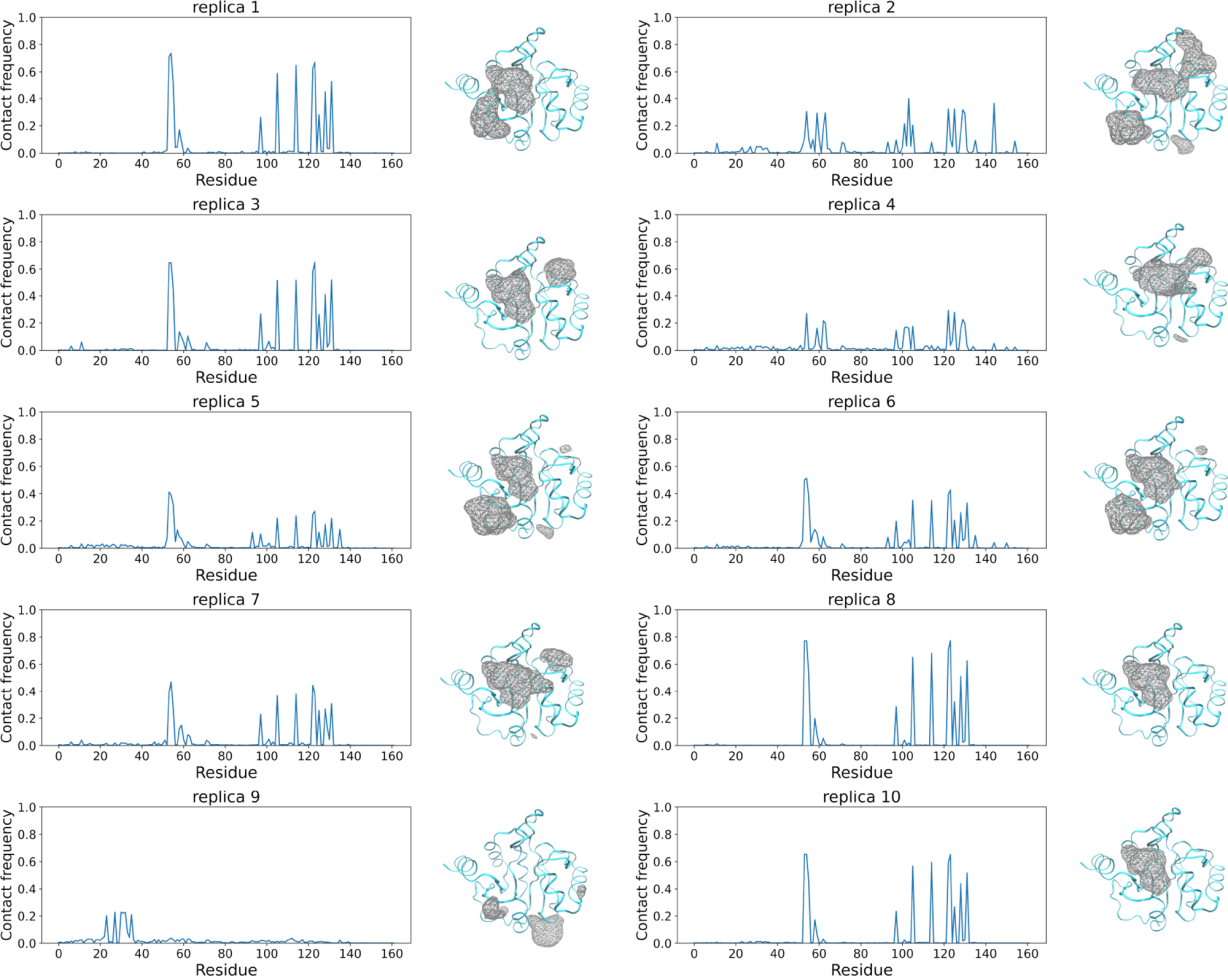
Tofacitinib (ligand)–ADRP (protein) residue contact frequency distribution plots for each “long” replica are shown adjacent to their respective ligand occupancy maps. The ligand–residue contact frequencies correspond to the fraction of frames in which a hydrophobic contact is formed between the ligand and a given protein residue. Occupancy maps of tofacitinib around the ADRP protein represent the isovalue surfaces (wireframe representation) rendered at the fractional occupancy of 0.03 across all frames of the simulation trajectory. In other words, they represent volumes of the simulation box where the ligand is likely to be found with 97% probability, that is in 97% of all trajectory frames.

Table 2 provides the number of binding sites sampled by the entire ensemble of 10 or 12 replicas for each system (10 for ADRP–tofacitinib complex and 12 for all other systems) in column 2. In the third column, it also includes the number of replicas that visit each binding site for each system. It is evident that not all sites are sampled in all simulations. There is substantial variation in the binding sites sampled both across replicas for each system, as well as across all systems studied. For instance, in the case of the ADRP–tofacitinib system, four different binding sites are sampled by 3, 4, 9, and 6 replicas, respectively. Comparing this behavior with that of the other four systems studied (all relatively bigger in size), we can clearly see that they differ in that the number of sites observed is much higher with the number of replicas visiting each site being smaller. Taking the example of the PLPro–GRL system, there are 15 different binding sites observed with each only sampled by a single replica for all but one site. Furthermore, 9 out of 12 replicas exclusively sample only a single binding site. This behavior is in contradiction to what we see for the ADRP–tofacitinib system exhibiting the extent of variation in sampling that may be observed across different systems using an ensemble of long independent simulations. The sampling behaviors of the other three systems fall between the two extremes discussed above. It should be noted here that, in the above analyses, a replica is considered to have sampled a given binding site only if its ligand fractional occupancy is ≥0.03 around that site. In other words, a replica is assumed to have sampled only those binding sites that appear in the volume occupancy maps (and have non-negligible peaks in the corresponding contact frequency distributions) displayed in Figures 2 and S1–S4. It is possible that a replica has visited other binding sites too for a very short period of time but such transient events are neglected in our analyses as such a binding process cannot be considered stable.

**Table 2:** Sampling Frequency of the Different Binding Sites Across All “Long” Independent Replicas^a^

system	no. of binding sites	no. of replicas visiting each site
ADRP–tofacitinib	4	3, 4, **9**, 6
PLPro–GRL	15	1, 1, 1, 1, 1, 1, 1, 1, 2, 1, **1**, 1, 1, 1, 1
3CLPro–93J	13	1, **8**, 1, 2, 1, 1, 1, 3, 3, 1, 1, 1, 1
3CLPro–RQN	9	7, 2, 1, **10**, 2, 1, 1, 1, 1
3CLPro–RQN (with LZE)	12	6, 4, 2, 2, **3**, **2**, 4, 3, 1, 2, 1, 1
3CLPro–LZE (with RQN)	8	6, **6**, **5**, 3, 4, 3, 4, 1

a The middle column shows the number of different binding sites sampled across all replicas for a given system. The last column shows an ordered set of the number of replicas visiting a given binding site for all sites in the middle column. The number in bold font corresponds to the experimental binding site. Note that this only captures whether a replica samples a given binding site at all. It does not take into account the amount of time spent at a given binding site by any replica. The total number of replicas is 10 for the ADRP system and 12 for all others.

There is also a non-negligible variation in sampling across replicas for each system studied. Taking the ADRP–tofacitinib system as an example, as already noted, four different binding sites (denoted as A, B, C, and D) have been sampled collectively by the ensemble of ten 10 μs long replicas. The crystallographic site (C) is sampled by 9 out of 10 replicas whereas all other sites are only located by a smaller number of replicas. There are two replicas (IDs 8 and 10) that exclusively sample site C, whereas two other replicas (IDs 2 and 5) sample all four sites. The remaining six replicas sample different subsets of the four binding sites in different combinations and proportions. It should also be noted that the sampling of the ligand around site C is quite different across each of the 9 replicas as quantified in the following paragraphs. Similar behavior applies to all other systems studied. One might be tempted to hypothesize that the number of replicas visiting a given binding site is a function of the binding free energy. However, based on our results, we can safely reject this hypothesis. For instance, ADRP bindings sites A, B, and D have very similar binding affinities, but the numbers of replicas visiting them vary. This behavior is even more pronounced for other systems studied (comparing Table 2 and Figure S6).

In order to provide a visual representation of the sampling variation discussed above, we have calculated the contact frequency distributions for individual replicas (refer to section 3 for details). Figure 2 displays contact frequency distributions of the ADRP–tofacitinib complex for each of the ten long (10 μs duration) simulations along with corresponding volume occupancy maps. It can be clearly seen that the signature frequencies of site C are visible in the contact frequency plots of all but one replica (only replica ID 9 does not sample the crystallographic site). Replica IDs 8 and 10 exclusively sample site C and hence display identical peak distributions, whereas other replicas have different peak distributions due to overlapping frequencies from other binding site samples. Similarly, replica ID 9 predominately samples site A, clearly showing the corresponding signature frequencies. Another replica that has a non-negligible peak at site A frequencies is replica ID 2 as is also confirmed by the corresponding volume occupancy maps. It should be noted that the magnitude (peak heights) of these signature frequencies for different binding sites are different across replicas. Similar figures with contact frequency distributions of the other systems studied have been included in the Supporting Information (Figures S1–S4) which all convey the same message as above.

##### 4.1.1.1 Quantification of Aleatoric Variability

In order to derive robust insights, it is essential that we quantify the extent of variability between the long replicas so as to determine their reproducibility. To achieve this, we compute pairwise Kolmogorov–Smirnov (KS) test statistics for each pair of the long replicas. The pairwise KS statistic has a range of 0 to 1, where 0 indicates that the two sample distributions being compared are sampled from an identical underlying distribution, and 1 indicates the converse case. We calculated pairwise KS statistics for 45 possible pairs of replica trajectories for the ADRP–tofacitinib complex. The resultant values fall between a wide range of 0.11 to 0.54. However, 40 of them are ≥0.15 and 37 are ≥0.2. The mean value of the KS statistic for all 45 pairs is 0.26. We also obtained corresponding *p*-values from pairwise KS statistics. A *p*-value of <0.05 signifies that the null hypothesis (that the two underlying distributions are identical) can be rejected with 95% confidence. This, in turn, means that there is a 95% chance that the two samples compared are drawn from different underlying distributions. We obtain a *p*-value of ≥0.05 for only 3 out of the 45 pairs of replicas (∼6.67%). Thus, 42 pairs (∼93.33%) indeed sample nonidentical regions of phase space. Table 3 contains relevant statistics (as discussed above for the ADRP system) for all systems which shows that the variability across replicas is prominent in all systems studied without exception at the microsecond time scale.

**Table 3:** Mean and Range of KS Statistics Values Across All Replicas for All Systems Studied^a^

system	mean	range	KS ≥ 0.2	*p*-value ≥ 0.05
ADRP–tofacitinib	0.26	0.11–0.54	82.2	6.7
PLPro–GRL	0.32	0.13–0.59	95.4	0
3CLPro–93J	0.31	0.15–0.61	97.0	0
3CLPro–RQN	0.26	0.11–0.52	78.8	1.5
3CLPro–RQN (with LZE)	0.33	0.10–0.66	86.4	1.5
3CLPro–LZE (with RQN)	0.23	0.12–0.50	75.8	0

a The number of KS values ≥ 0.2 (an arbitrary threshold) as well as the number of *p*-values ≥ 0.05 (in percent terms).

**Figure 3:**
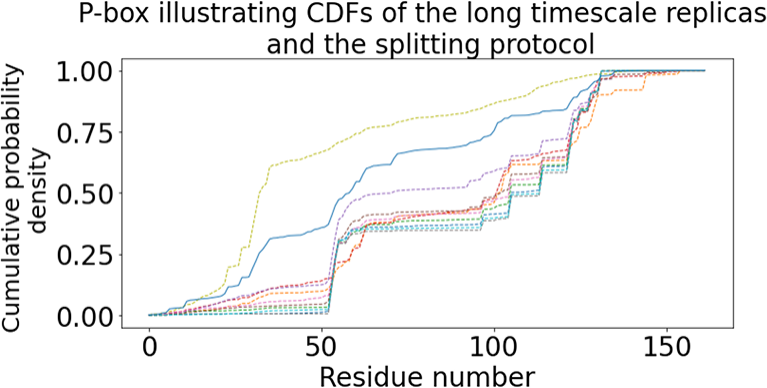
Cumulative density functions (CDFs) of the contact frequency distributions for all “long” replicas (dashed lines) as well as concatenated splitting protocol trajectories (solid line) for ADRP–-tofacitinib system. The width of the p-box so generated indicates the extent of variability across “long” replicas compared against the splitting protocol.

Figure 3 displays the cumulative density functions (CDFs) of ligand–residue contacts for all ten replicas of the ADRP system. Constructs known as p-boxes (regions between two extreme CDFs) are often used to visualize how the distribution of outcomes is controlled by aleatoric and epistemic uncertainty.^79^ It is clear from Figure 3 that the p-box generated by ten “long” independent replicas has a wide range, another representation of the extensive variation of sampling across replicas. Figures displaying the CDFs of ligand–residue contacts and corresponding p-boxes for all other systems have been included in the Supporting Information (Figure S5) with identical observations.

The above findings establish beyond doubt the nonreproducibility of long trajectories for moderately sized protein–ligand systems at least for simulations of duration up to 10 μs. They confirm that it is far-fetched to draw final conclusions on the true nature of a system from an individual MD simulation as two independent trajectories would sample different regions of the phase space for reasonably long temporal durations when starting from different initial conditions.^15^ Indeed, this is a direct reflection of the chaotic nature of molecular dynamics simulation and, from a theoretical standpoint, shows that individual 10 μs trajectories can never be used to determine equilibrium behavior. In fact, equilibrium is meaningful only for ensembles of trajectories which manifest the required dynamical instability. While individual trajectories are time reversible, the approach to equilibrium is a probabilistic property of ensembles which requires the dynamics to be chaotic.^15^

##### 4.1.1.2 Variability in Free Energy Estimates

Free energy is a thermodynamic observable of importance for protein–ligand complexes in the drug discovery context. Therefore, we also look at the extent of variation in free energy estimates obtained using independent “long” replicas of MD simulations. We used Δ*G*_direct_ as a measure of absolute binding free energy which was originally developed by De Jong et al.^75^ and later applied to 10 μs trajectories by Pan et al. in 2017^21^ (details in section 3). In the present work, we demonstrate that Δ*G*_direct_ varies substantially between separate independent long time scale replicas, and hence once again individual “long” simulations do not provide reliable binding free energy estimates. The salient point here is that, contrary to received wisdom in the literature on molecular dynamics, averaging over an individual long time scale simulation is not equivalent to averaging over an ensemble of simulations. Indeed, thermodynamic quantities arise from ensemble averaging in statistical mechanics, and unless one averages over a time scale on the order of a Poincaré recurrence, a one-off MD trajectory will produce the wrong results.^15^ Compounding this, a one-off simulation does not provide the means to compute precise results or conduct meaningful uncertainty quantification.

**Figure 4:**
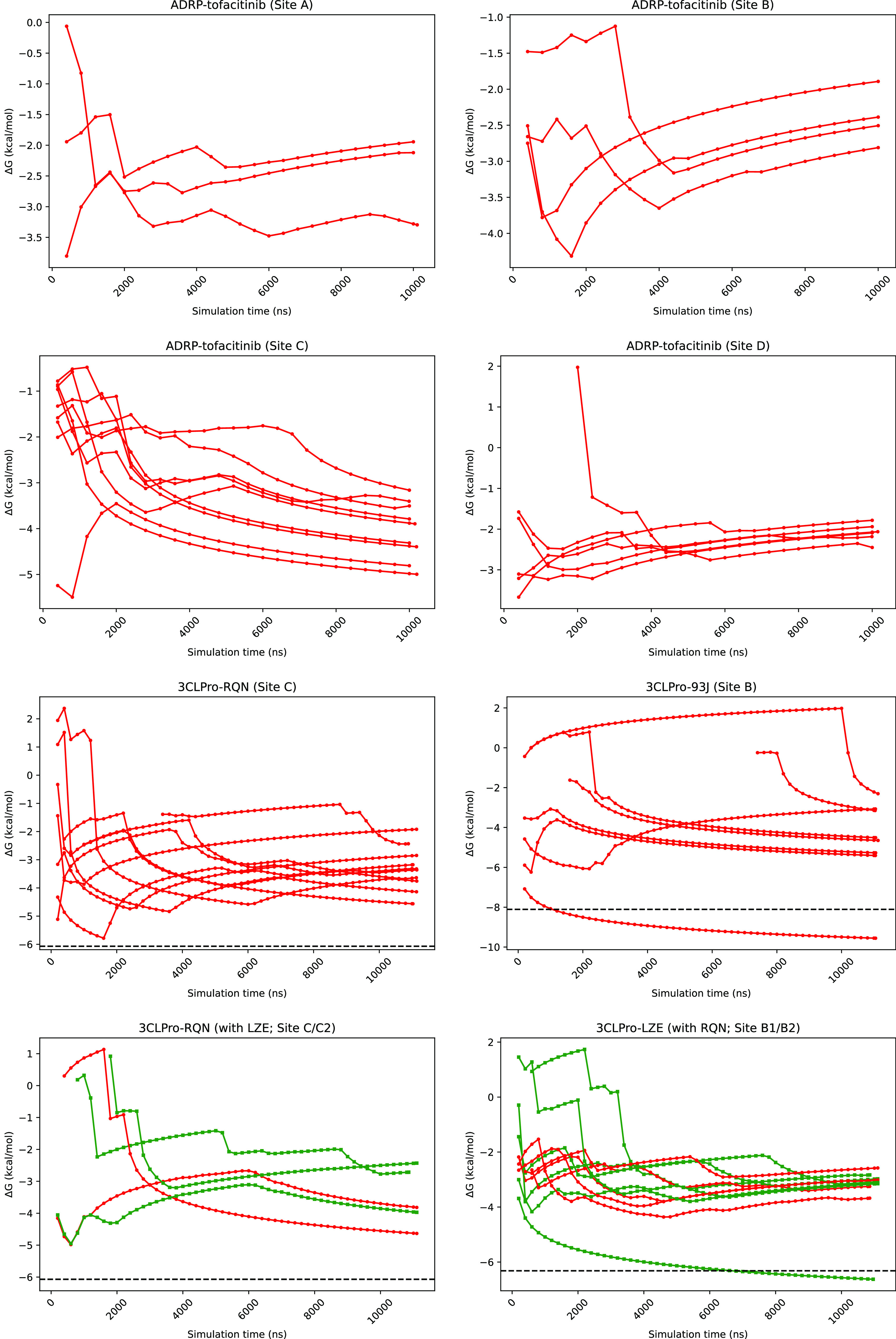
Running averages of Δ*G*_direct_ for tofacitinib binding to ADRP at all of the identified ADRP binding sites (top two panels) and for the experimental binding sites of 3CLPro systems (bottom two panels) for all ten or twelve 10 μs trajectories. The horizontal black dashed lines in the plots of 3CLPro systems correspond to the respective experimental binding affinities.

Figure 4 shows the running averages of Δ*G*_direct_ for all four binding sites of the ADRP system (top two rows) as well as for crystallographic sites of all 3CLPro systems (bottom two rows) from all replicas that sample them. The inter-replica variation is clearly visible from these plots for all systems. This variability shows that results obtained from individual “long” trajectories are not reproducible or precise. In fact, it is evident that the Δ*G*_direct_ estimator does not even converge for some replicas, even though it does for others. This itself is a source of uncertainty and a strong motivation to perform ensemble simulations. Nevertheless, even when all or most replicas do converge, their predictions vary non-negligibly. Figures displaying running averages of Δ*G*_direct_ for all binding sites of all systems studied have been included in the Supporting Information (Figure S6). All of them show behavior similar to that discussed above in terms of Δ*G* variability. Table 4 includes the mean Δ*G*_direct_ values along with error bars for the most frequently visited binding site (which is not the experimental binding site for PLPro–GRL and 3CLPro–RQN (with LZE)) across all replicas for each system. It also provides the spread (that is, the difference between the two extreme values) for each such binding site which is around 2–3 kcal/mol for most cases but can be as high as 7 kcal/mol (for instance 3CLPro–93J). Another point worth noting from Figures 4 and S6 is that the first binding event occurs at varying time durations across ensemble members as captured by the different onset simulation times of the running average plots. This confirms that the dynamical behavior has substantial variability at the microsecond time scale for molecular dynamics, just as it does for shorter time scales.

**Table 4:** Mean and Spread (That Is, Difference between Extreme Values) of Δ*G*_direct_ Across All Replicas for the Binding Site That Is Visited by the Most Number of Replicas for Each System Studied^a^

system	mean	spread (no. of replicas)
ADRP–tofacitinib	–4.03(0.20)	1.83(9)
PLPro–GRL	–3.47(0.27)	0.77 (2)
3CLPro–93J	–4.74(0.74)	7.26 (8)
3CLPro–RQN	–3.32(0.23)	2.64 (10)
3CLPro–RQN (with LZE)	–3.95(0.32)	2.16 (6)
3CLPro–LZE (with RQN)	–3.63(0.55)	3.80 (6)

a Note that such a binding site is not always the experimentally determined one. Error bars are the standard errors. All values are in kcal/mol.

**Figure 5:**
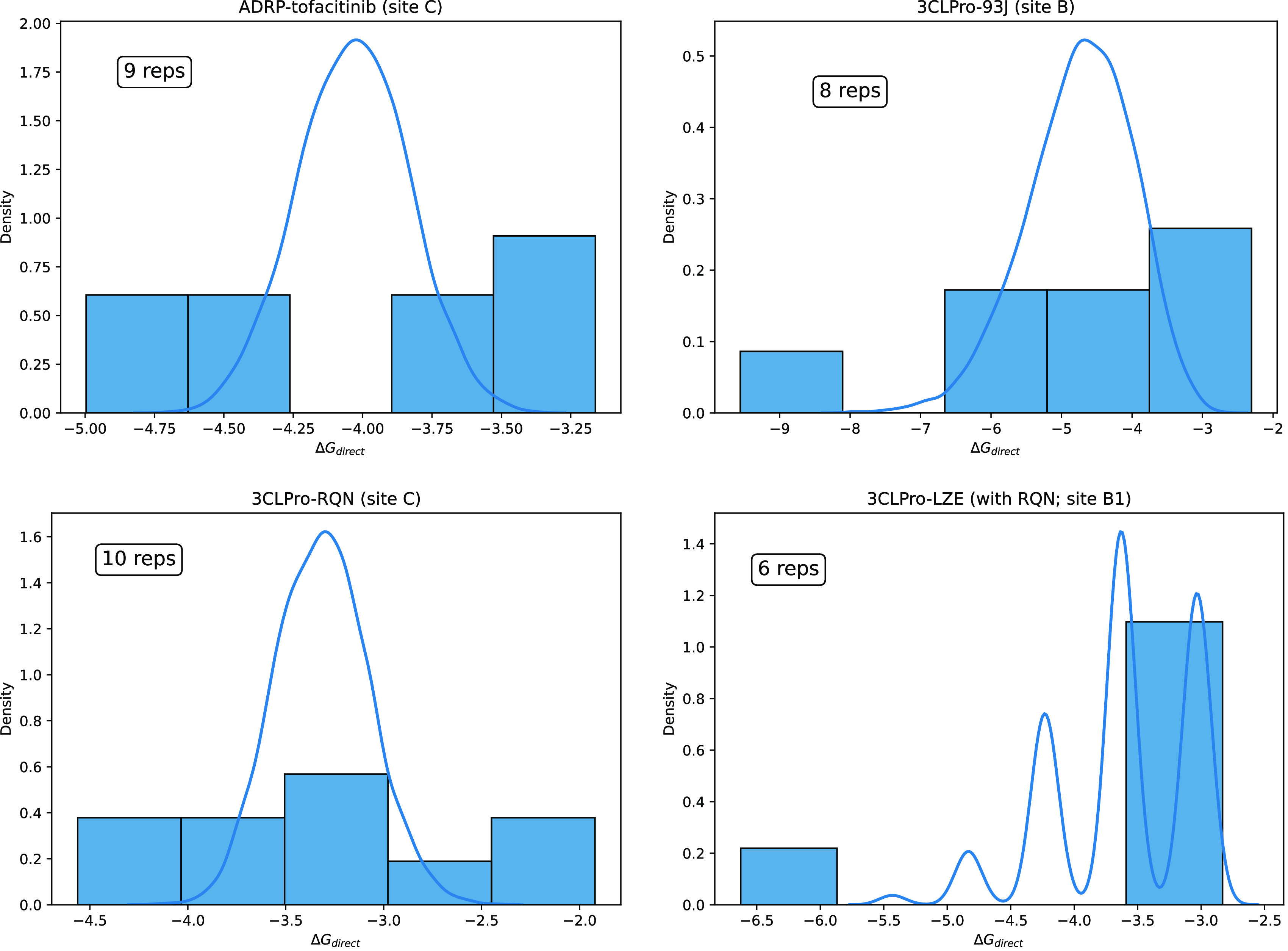
Probability density functions of Δ*G*_direct_ values using 5 bins as well as sampling distributions of mean direct free energies (⟨Δ*G*_direct_⟩) obtained with bootstrapping (5000 resamples) for four of the systems studied at their respective crystallographic binding sites. Bar plots display density histograms of the set of final Δ*G*_direct_ values from all replicas that sample the respective bindings sites, which constitutes the original sample used for bootstrapping, whereas solid lines represent kernel density estimations of corresponding sampling distributions of mean obtained using bootstrapping. Sizes of the original samples are shown in the text boxes within each plot. The data suggests that there may be non-Gaussian behavior in the underlying distribution. The *x*-axis is expressed in kcal/mol.

To obtain meaningful estimates of Δ*G*_direct_, we must take into account the results from all members of an ensemble. To do this, we employ bootstrapping to obtain sampling distributions of the mean for Δ*G*_direct_ by resampling 5000 times with replacement. The original sample used for such analysis is the ensemble of Δ*G*_direct_ values from all replicas that sample a given binding site. The probability density functions of the original sample as well as the sampling distributions of means so obtained are displayed in Figure 5 for a selection of systems studied. As we have shown in previous studies for relatively short duration MD trajectories, it is possible that the underlying free energy distributions may be non-Gaussian whereas the corresponding bootstrapped distributions approach the Gaussian functional form with increasing sample size as a consequence of the central limit theorem.^18,60,61,80,81^Figure 5 provides evidence of similar behavior in case of “long” MD simulations as well, although given the small sample sizes (as shown in the inset) not all bootstrapped distributions are Gaussian either. To be sure, an ensemble of size ≤10 is far too small to draw definitive conclusions on the true form of the underlying distribution. To ensure convergence of Δ*G*_direct_, it would be necessary to determine the change in the bootstrapped value of Δ*G*_direct_ as a function of the number of replicas. Upon convergence, the estimate for the binding free energy could be classified as reliable and reproducible.

The crucial idea here is that to even begin to generate reproducible estimates for Δ*G*_direct_, running ensembles of simulations, irrespective of their length, is an imperative. Interestingly, the non-normal nature of free energy distributions implies more frequent occurrence of outliers than would be expected with normal distributions that necessitates relatively more data in order to obtain reliable estimates. Since the variability that exists across replicas within an ensemble of simulations is caused by the intrinsically chaotic nature of MD simulations, these principles will apply to the calculation of any MD derived macroscopic expectation value.

#### 4.1.2 Biased versus Unbiased Sampling

In the previous section, we have described results from unbiased MD simulations and shown that the sampling may vary substantially on repeating a simulation. In this section, we include results from the biased simulation protocol named the “splitting protocol” which involves biasing the sampling of phase space toward sites of interest (described in detail in section 3). It should be noted that, in principle, such splitting steps can be continued further until the desired level of sampling has been achieved (for instance, if there is a substantial variation in the binding poses across subreplicas that need to be explored further, and so on).

In the case of ADRP–tofacitinib, a highly multimodal distribution is observed across the initial 20 200 ns replicas with multiple binding sites explored. All four binding sites are identified while each replica possesses a unique distribution of tofacitanib–residue contact frequencies. For the ADRP system, the subreplicas were initiated from the final frame of replicas 1, 9, 14, 15, and 20, where the ligand was positioned at binding sites A, B, C, and D.

**Figure 6:**
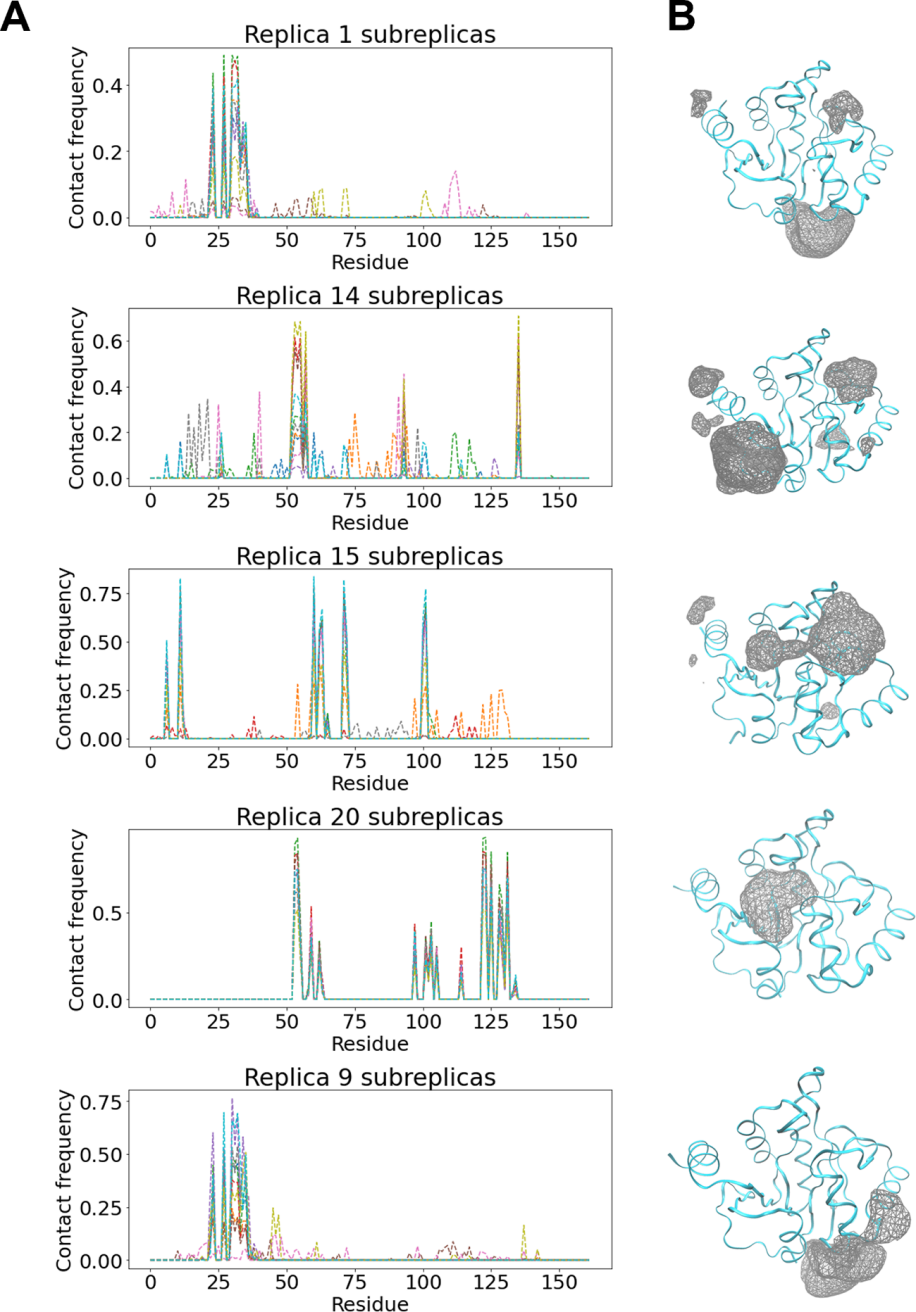
ADRP–tofacitinib complex with splitting protocol: (A) Distribution of ligand–residue contact frequencies for each set of subreplicas. (B) Volume occupancy maps of the ligand around protein rendered at an isovalue of fractional occupancy 0.03. For each set of subreplicas, the wireframe isosurface represents the area of the simulation box where the ligand is likely to be found with 97% probability.

As noted earlier, site C is the crystallographically defined site of ADP ribose and has the most negative binding affinity for tofacitinib. This relatively high thermodynamic stability at site C is also reflected in the occupancy maps and ligand–residue contact frequency distribution plots of subreplicas initiated from the end frame of replica 20 as shown in Figure 6. The ligand possesses a well-defined pose across all replicas when initiated from site C (subreplicas of replica 20). This is contrary to the behavior seen when initiating subreplicas from the ligand located in other binding sites where the ligand explores multiple sites over each set of subreplicas as also evident from Figure 6. Overall, this provides evidence that tofacitinib would act to competitively inhibit the protein.

The important thing to note here is that the aleatoric nature of MD has been utilized to our advantage to substantially accelerate the exploration of phase space by introducing appropriate bias to the sampling compared to a single simulation of the duration given by the aggregate time of all runs under the splitting protocol in a much shorter wall-clock time. In the following paragraphs, we further substantiate this point by comparing the results from biased and unbiased sampling.

**Figure 7:**
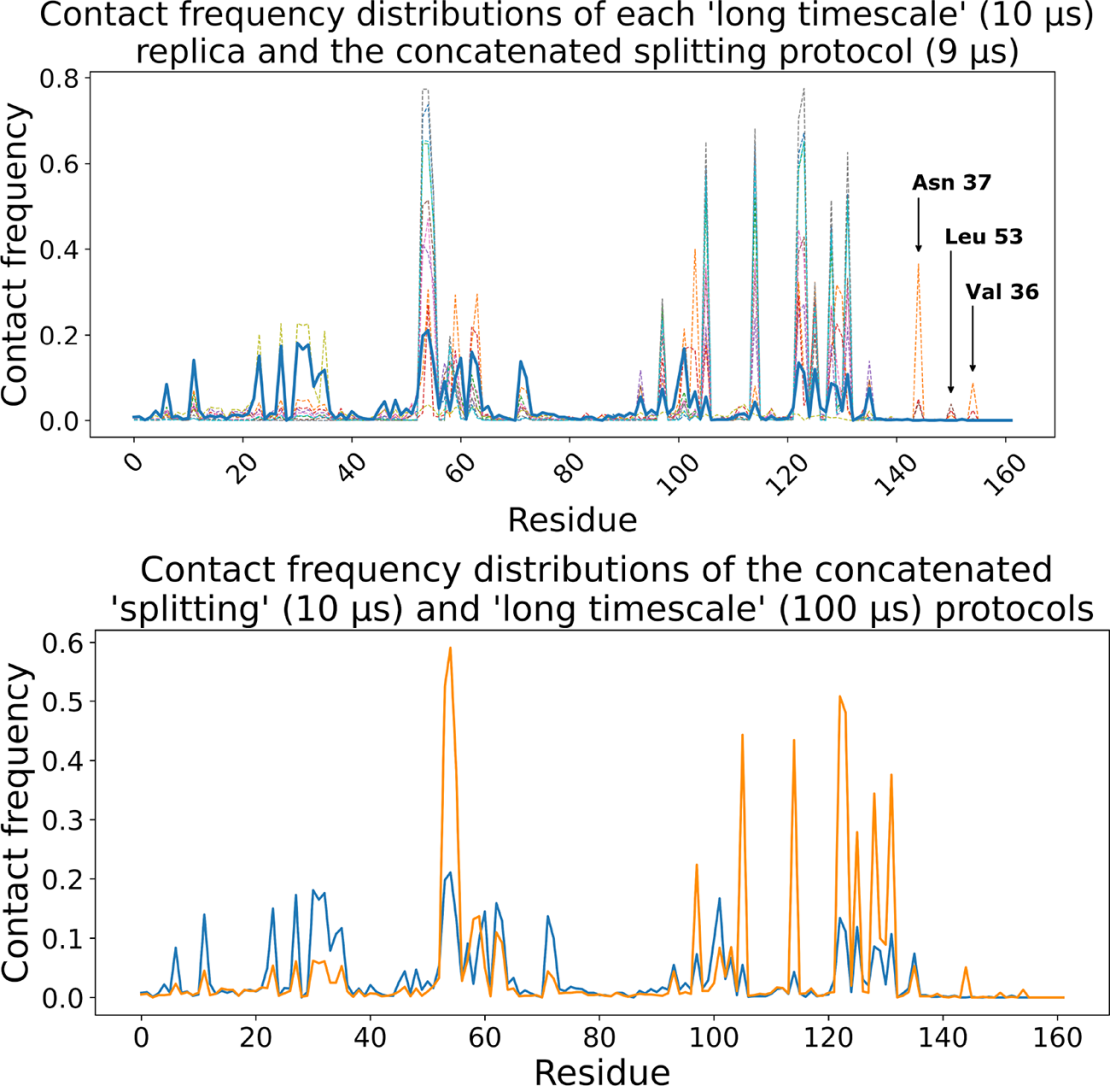
Contact frequency distributions of all “long” (10 μs) replicas individually (dashed lines in the top panel) as well as concatenated (solid orange line in the bottom panel) compared to that of the splitting protocol (9 μs) (solid blue line) for ADRP–tofacitinib complex.

First of all, we compare the contact frequency distribution of the aggregated (biased) sampling using the splitting protocol (9 μs) with those from all the individual unbiased sampling from the 10 long simulations (10 μs each). Figure 7 (top) displays such comparisons for ADRP–tofacitinib complex (refer to Figure S7 in the Supporting Information for all other systems). It is evident that each 10 μs replica samples a mere subset of possible binding sites explored across the 9 μs of the splitting protocol. The only exception to this general observation are the contacts of the ligand with residues 140–160 that are exclusively observed in long simulations. We will discuss this exception below. The significant difference in sampling between the splitting protocol and each individual long replica is quantified with two-sample KS statistics. For ADRP system, KS statistics varies between 0.25 and 0.36 with an average of 0.31, and the average of all corresponding *p*-values is 1.37 × 10^–5^. Table 5 shows mean and range of KS statistics values for other systems. They fall in a similar range going as high as 0.6 and as low as 0.13 in some cases.

**Table 5:** Mean and Range of KS statistics Values Comparing Splitting Protocol with Each Long Replica for All Systems Studied^a^

system	mean	range	KS ≥ 0.2
ADRP–tofacitinib	0.31	0.25–0.36	100
PLPro–GRL	0.30	0.21–0.47	100
3CLPro–93J	0.32	0.21–0.48	100
3CLPro–RQN	0.21	0.14–0.39	58.3
3CLPro–RQN (with LZE)	0.42	0.13–0.6	83.3
3CLPro–LZE (with RQN)	0.26	0.18–0.44	83.3

a The number of KS values ≥ 0.2 (an arbitrary threshold) is given in percent terms.

Figure 3 displays a p-box for the contact frequency distributions for the splitting protocol as well as all long replicas separately for the ADRP system (and Figure S5 for other systems). It clearly shows that the bounds on the cumulative probability of the ligand contacting a given residue across the full set of simulations furnish a clear visualization of the aleatoric uncertainty that is associated with the ligand–residue contact frequency across all simulations.

On carefully observing Figure 7 (top), it can be noted that when considering all long trajectories in aggregate, we are able to recover all ligand–protein interactions which are identified across the splitting protocol. To see this more clearly, we plotted the ligand–residue contact frequency distributions for aggregated sampling time from both the splitting protocol (9 μs in total) as well as unbiased sampling (100 μs in total) for the ADRP system in Figure 7 (bottom). Here we see that the concatenated trajectory reproduces all modalities which occur across the splitting protocol, albeit with different statistical weights. Incidentally, if our aim is to simply explore all possible binding sites and dominant poses within those sites, the unbiased long time scale protocol is far less efficient than the splitting protocol which achieves this aim in an aggregate of 1 day and 7 h of wall clock time rather than 43 days 2 h of wall clock time required with the former. However, care must be taken when thermodynamic quantities need to be evaluated/predicted using the splitting protocol as the biased sampling leads to biased weights of the microstates sampled that may affect the averages obtained.

Nevertheless, there are advantages to performing an ensemble of long simulations rather than the splitting protocol. Namely, there are key poses and contacts identified during the long time scale protocol which are highly unlikely to be observed by the shorter time scale splitting protocol. In Figure 7 (top), we see three tofacitinib–ADRP contacts which were observed during the long time scale protocol but not during the splitting protocol. These contacts occur with residues Asn37, Leu53, and Val36. Upon inspection, we find that all three residues are buried deep within site C (the crystallographically determined binding site). This indicates that a long duration of wall time is typically required in order to explore these “rare” poses as access is required to more buried regions of the ADRP active site. It should be noted that such contact frequency peaks corresponding to conformations that are exclusively sampled in the “long” trajectories are present much more prominently in case of all other systems studied (see Figure S7).

Until now, we have discussed the variation across long time scale MD trajectories and emphasized that ensemble simulations are necessary for UQ irrespective of the duration of simulation. However, as already discussed in section 1, several accelerated sampling protocols (including the splitting protocol employed in this study) that are based on performing “ensembles” are also expected to exhibit similar variation and would require performing ensembles for UQ. We have already shown this for replica exchange methods in some of our previous works.^17,56^ Nevertheless, this aspect has not been addressed adequately in the literature for other accelerated sampling protocols as the reported errors for such methods are all derived from the data generated from a single execution of the protocol, but never from ensembles comprising multiple instances. One reason for this shortcoming might be the computational cost associated with all these methods. We hope to return with a subsequent study where we will discuss this issue systematically.

#### 4.1.3 Free Energy Methods: Direct versus ESMACS

**Figure 8:**
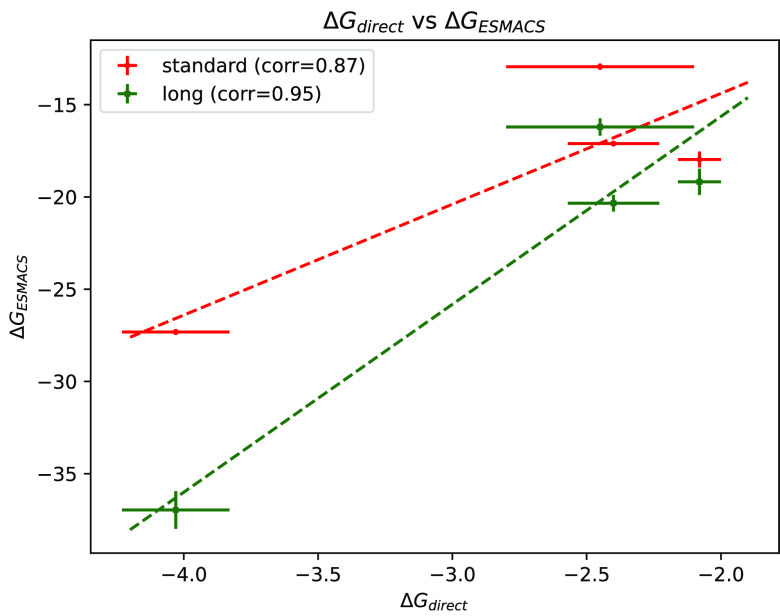
Δ*G* values obtained from different free energy protocols: Δ*G*_direct_ compared against Δ*G*_ESMACS_ using both the standard ESMACS protocol as well as that using bound conformations extracted from “long” trajectories. “corr” denotes the Pearson’s correlation coefficient. Dashed lines denote the best fit lines for each plot. All values are in kcal/mol.

In this section, we have compared free energies obtained from different free energy protocols. We have already seen Δ*G*_direct_ results for the different systems in previous sections. Now, we directly compare them to Δ*G*_ESMACS_ results obtained through the ESMACS protocol for the ADRP–tofacitinib system. The standard ESMACS protocol (denoted as “ESMACS-s” which involves performing an ensemble typically of 25 MD simulations of 4 ns duration starting from a chosen conformation) has been extensively applied to a diverse range of protein–ligand systems and shown to rank ligands with very high precision.^66^ In this study, we chose the most stable binding pose (the one with the least RMSD) from the different subreplicas of the splitting protocol at each binding site as the starting structure for our standard ESMACS calculations. Table S1 and Figure 8 show a comparison of Δ*G*_direct_ and Δ*G*_ESMACS-s_. We find that both methods achieve strongly correlated results with a correlation coefficient of 0.87.

The direct method involves a much larger amount of sampling as compared to ESMACS-s which involves performing short MD simulations of only a few nanoseconds duration. It is, however, notable that ESMACS-s is still able to obtain almost identical ranking of ligand–protein complexes with such little sampling, making it a much more efficient method when accuracy is not necessary. Nevertheless, it is well-known that ESMACS-s results depend heavily on the initial binding pose/structure of the ligand–protein complex being studied due to the short duration of simulations; this can be a drawback in some cases where the initial structure is not known correctly. In such cases, ESMACS-s is not so useful as resultant Δ*G* values may vary substantially (see specific details in the Supporting Information). On the other hand, due to substantially more sampling, the direct free energy method is expected to overcome this drawback. In this study, we have performed ESMACS calculations using all “bound” conformations (as defined so during Δ*G*_direct_ calculation) extracted from all “long” trajectories such that the ensemble averaging is performed across all replicas that sample a given binding site (denoted as “ESMACS-l”). Free energies so obtained are expected to be free from the dependence on starting structures and better correlated with Δ*G*_direct_. This is evident in Figure 8 where Δ*G*_ESMACS-l_ are consistently more negative than Δ*G*_ESMACS-s_ and have a higher correlation coefficient of 0.95. This is because all the different binding poses sampled during the long duration of simulations have been taken into account with appropriate weights. The key conclusion is that it is sufficient to apply ESMACS-s for ranking ligands based on their binding affinities when the starting structure is confidently known; otherwise extensive sampling becomes necessary.

### 4.2 Allosteric Modulations in Main Protease

In this section, we present important allosteric mechanisms in the main protease of SARS-CoV-2. Using our ensemble-based methods, we were able to reliably recognize allosteric modulations hitherto unknown. We elucidate binding cooperativity between RQN and LZE ligands such that the presence of LZE affects the binding interactions of RQN at its active site and vice versa. Such modulations also provide an explanation for the inhibitory effect of LZE. Thanks to our ensemble approach, we are able to confidently state that such effects do not affect RQN’s binding affinity. In addition, we also discovered an unknown mechanism of action of RQN ligand which has been shown to act by binding at the substrate binding site. We show that RQN binds at a binding site away from the substrate binding site triggering the rotation of domain III of the main protease. We also show that this rotation is prevented by the binding of LZE. Below we discuss all the above novel discoveries in detail.

#### 4.2.1 Cooperative Binding of RQN and LZE

Ligand RQN binds to the active binding site of the 3CLPro target protein whereas LZE binds to the allosteric binding site II.^63^ In this study, we have performed simulations that contain both RQN and LZE ligands binding to the 3CLPro target at the same time. Therefore, we discuss the observed effect of the presence of LZE on the binding of RQN ligand by comparing the results from this system with those from the system containing only RQN. First of all, the presence of LZE does not affect the value of Δ*G*_direct_ for RQN binding with 3CLPro. The respective values in the presence and absence of LZE are −3.04 ± 0.39 kcal/mol and −3.32 ± 0.23 which are statistically the same. However, the important thing to note here is that the respective spreads (difference between extremes) in Δ*G* values for these systems are 1.54 kcal/mol (ranging from −3.97 to −2.43 kcal/mol) and 2.64 kcal/mol (ranging from −4.56 to −1.92 kcal/mol) which are both much larger than the difference between their mean Δ*G* values. This indicates the importance of performing ensembles in order to obtain statistically robust and reliable conclusions. For instance, taking the opposite extremes of Δ*G*_direct_ values for both systems, we could have obtained differences of either −2.05 or 2.13 kcal/mol in the presence and absence of LZE, respectively, leading to diametrically opposite conclusions on its effect on the binding of RQN. But on performing ensemble simulations we are able to state with confidence that no statistically significant effect has been observed.

**Figure 9:**
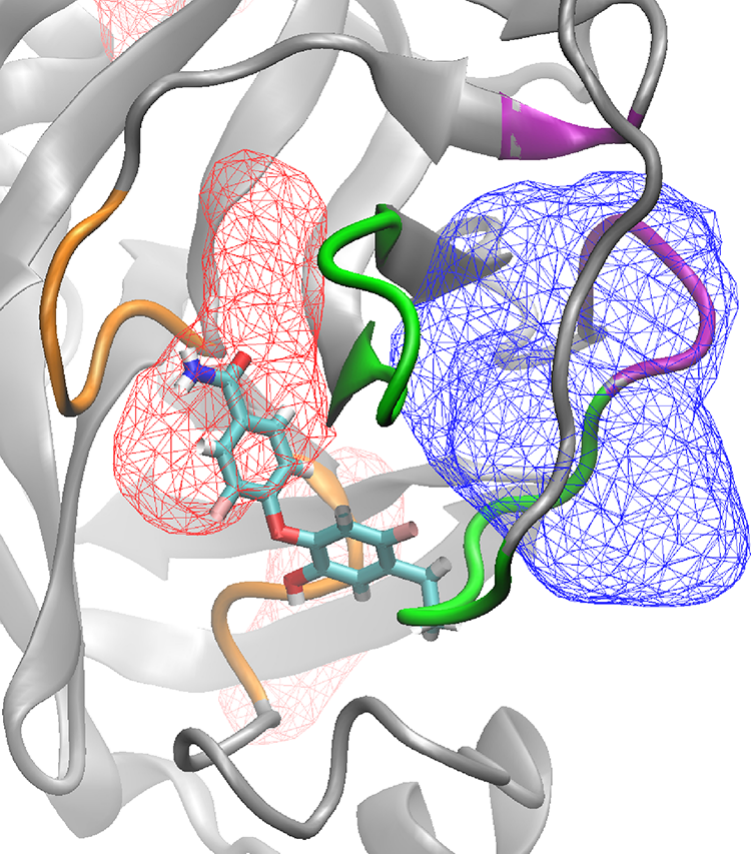
Effect of the presence of LZE on the binding of RQN. The experimental binding site (red) as well as the alternate binding site observed (blue) are shown in terms of volume occupancy maps using wireframe isosurfaces at isovalue 0.3. The crystallographically determined binding pose has also been shown in the “bonds” representation. Loops 167–172 and 186–191 (shown in green) are common to both binding sites. Loops 40–43 and 141–145 (shown in orange) are exclusive to the experimental binding site, whereas loop 182–185 and residues 134–135 are exclusive to the alternative site observed.

Another important effect that has been observed is the emergence of a new binding site for RQN (denoted as C2), very close to the experimentally observed binding site (denoted as C), when binding to 3CLPro active site in the presence of LZE. Experimentally, it has been shown that the binding of LZE at allosteric site II displaces the loop 153–155 such that the C_α_ atom of Tyr154 moves 2.8 Å, accompanied by a conformational change of Asp153.^63^ This loop is connected to loop 167–172 through a β-sheet strand 156–166 which is expected to cause a shift in the former as well. We have quantified this effect using our simulations. Five replicas, wherein LZE binds to site B1, were processed to extract only the frames in which LZE is indeed bound to the said binding site and RMSDs calculated for loops 153–155 and 167–172 at each frame. Figure S8 displays the time series of both these RMSD values for all replicas. It can be clearly seen that the RMSDs of both loops are correlated with both increasing/decreasing at the same time. This provides evidence for the displacement of loop 167–172 through LZE binding. Figure 9 displays binding sites C (red) and C2 (blue) for RQN in the form of observed volume occupancy maps. Binding sites C and C2 have loops 167–172 and 186–191 in common (shown in green). Therefore, the binding of LZE at allosteric site II brings about conformational changes to the active site and creates enough space to let RQN bind at a slightly different location, very close to the original site. It appears that such a change does not have any substantial impact on the binding interactions of RQN with the residues of site C, thereby not affecting its binding affinity. However, its sampling frequency is certainly affected such that, in the absence of LZE, it is sampled by 10 out of 12 “long” replicas, whereas in its presence, it is sampled only by 3 out of 12 “long” replicas. On the other hand, site C2 is sampled in 2 out of 12 “long” replicas (exclusively in the presence of LZE). A similar effect has been observed in the case of LZE with RQN present such that a new close-by binding site (denoted as B2) is sampled along with the crystallographically determined binding site (denoted as B1). Table 2 and Figure 4 include both such binding sites (for both RQN and LZE in the presence of each other) as experimental binding sites and display results accordingly.

##### 4.2.1.1 LZE Inhibition Mechanism

It is noteworthy that the displacement of loop 167–172 induced by LZE binding also provides a possible mechanism of its inhibitory effect. It is known that 3CLPro is only active as a dimer.^82,83^ After its dimerization and activation, the N finger of each monomer interacts with Glu166 of the other to perform the catalytic activity. Similarly, His172 is involved in forming a salt-bridge with the other monomer essential for dimerization.^82^ Thus, the displacement of loop 167–172 negatively affects the dimerization of 3CLPro which explains the inhibitory effect of LZE binding.

#### 4.2.2 Alternative Mechanism of Action of RQN Inhibition

It is known that RQN (also known as MUT056399) binds at the substrate binding site of 3CL protease (also known as main protease) and inhibits viral replication by blocking access to the catalytic site consisting of Cys145 and His41.^63^ In this study, we have uncovered another mechanism of action of RQN when binding to 3CL protease through which it is able to inhibit the catalytic activity of the main protease. This involves binding to a different binding pocket (denoted as A1). Figures S9–S11 display RMSD time series of Arg298 for all 12 “long” replicas for all three 3CLPro complexes studied (binding to 93J, RQN, and RQN + LZE). There are sections of trajectories in several replicas where the RMSD value shoots up to values as high as 12 Å or more. Interestingly, this behavior is only observed when RQN is present in the system (that is, we do not see such high RMSD values in any replica of the 3CLPro–93J complex). Moreover, in the case of the 3CLPro−RQN−LZE system, this behavior is found exclusively in replicas where LZE does not bind at the binding site B1 (see Figure S9 for details). In other words, LZE binding at site B1 prevents such a large increase in Arg298 RMSD. Below we explain both such behaviors in detail.

**Figure 10:**
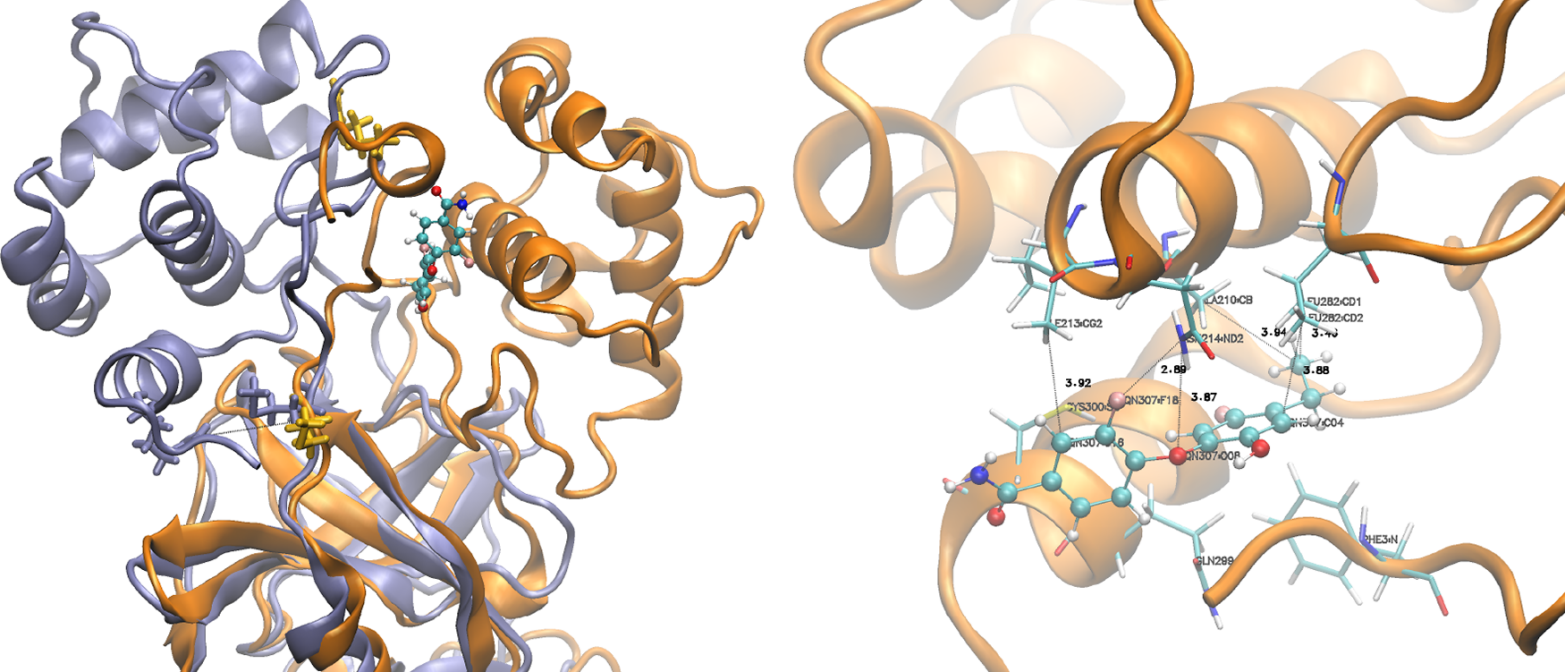
(left) Comparison of the first (purple) and the last (orange) frame of replica 9 of the 3CLPro–RQN–LZE system (see figure S9) with their domains I and II (residues 1 to 197; the bottom half) aligned. Protein is displayed as ribbons whereas residues Met6 and Arg298 of each frame (also colored accordingly) are shown as sticks. The hydrogen bond between Arg298 and Met6 in the first frame (purple) is shown as a dashed black line. Ligand RQN (bound only in the last frame) is displayed as lines (bonds) and balls (atoms). It is clear that domain III (residues 198 to 303) of the protein rotates leading to a substantial conformational change. (right) Interaction profile of ligand RQN with different protein residues around it (a magnified version of the binding site from the left panel) with key protein residues displayed additionally as sticks and various interactions as dashed black lines.

In order to understand the large RMSD values for Arg298, we compare the first and the last MD snapshot of replica 9 of the 3CLPro–RQN–LZE system (see Figure S9) which have very different RMSDs. Figure 10 (left panel) compares the protein conformations in both of these snapshots. It appears that domains I and II (residues 1 to 197) are still well-aligned in both conformations, although domain III has rotated (still keeping its intradomain conformation intact). This rotation leads to a substantial change in protein conformation and a corresponding large increase in Arg298 RMSD. This rotation is similar to the one experimentally reported to be triggered by R298A mutation.^84^ The Arg298 residue is known to play a key role in stabilizing the relative position of N finger and domain III that is necessary for the dimerization of the main protease. The formation of a hydrogen bond between the NH_2_ of Arg298 and the backbone oxygen of Met6 has such a stabilizing effect (as shown by purple residues in Figure 10). Incidentally, RQN binding to site A1 (as shown in the left as well as right panel of Figure 10) requires the ligand to move between the N finger and domain III that creates a gap between Arg298 and Met6 (as shown by yellow residues in Figure 10). This leads to breaking of the hydrogen bond between these residues leaving domain III free to move causing the rotation as observed on mutating Arg to Ala experimentally. Such a rotation destabilizes the interdomain structure of the main protease rendering it incapable of dimerizing. It is well-known that dimerization is important for the catalytic activity of 3CL protease.^84^ Thus, we have discovered a novel mechanism of inhibition by RQN/MUT056399 without its binding to the substrate binding site.

**Figure 11:**
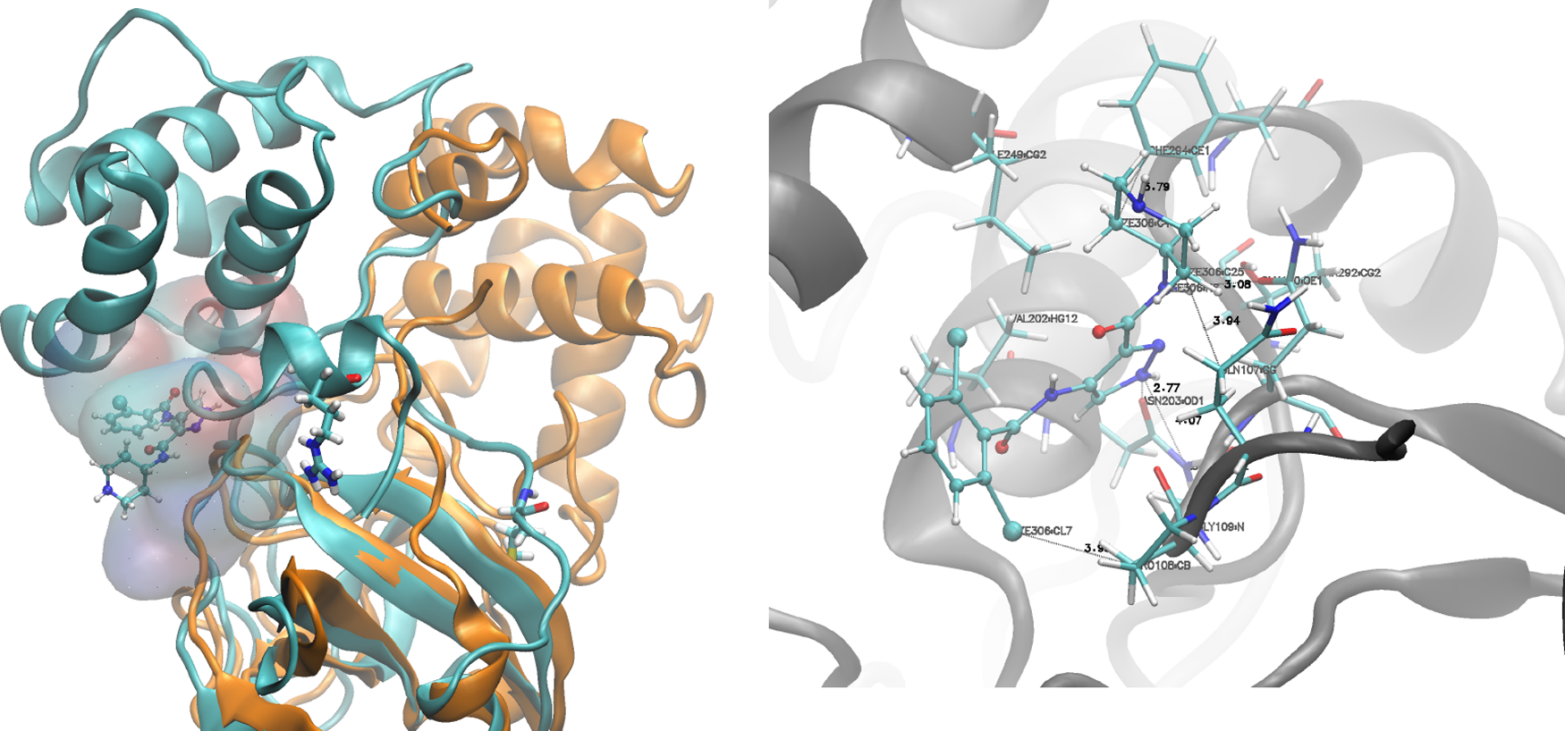
(left) Comparison of the last frame of replica 7 (cyan) and the last frame of replica 9 (orange) of the 3CLPro–RQN–LZE system (see Figure S9) with their domains I and II (residues 1 to 197; the bottom half) aligned. Protein is displayed as ribbons. Residues Met6 and Arg298 are shown as sticks whereas ligand LZE is displayed as balls (atoms) and lines (bonds) for replica 7. It is clear that the rotation of domain III (residues 198 to 303) observed in replica 9 does not take place in replica 7 with LZE binding at site B1. (right) Interaction profile of ligand LZE with protein (a magnified version of the binding site from the left panel) with key protein residues displayed additionally as sticks and various interactions represented as dashed black lines.

Another interesting observation from our simulations is that LZE binding at site B1 prevents this rotation of domain III. In order to understand this behavior, we compared the last frame of replica 9 (with domain III rotated) with the last frame of replica 7 (no rotation). Figure 11 (left panel) displays the comparison of these two conformations (replica 9 in orange and replica 7 in cyan). Arg298 and Met6 are also shown for replica 7, and it is clear that they are too far apart to form a hydrogen bond. Nevertheless, domain III does not rotate at all in replica 7 unlike replica 9. The proposed explanation here is that the binding of LZE at site B1 (shown as a surface in Figure 11 (left panel)) provides interdomain stability in lieu of the Arg298–Met6 interaction. As shown the right panel of Figure 11, LZE interacts strongly with several residues from both domain I/II (107–110) and III (202, 203, 249, 292, 294), thus holding them together and preventing the rotation of domain III even in the absence of the Arg298–Met6 hydrogen bond.

## 5 Conclusions

The current consensus in the field of molecular dynamics simulation is that increasing the length of a single simulation leads to improvement in the accuracy and precision of calculated expectation values.^85−87^ On the basis of chaos theory and the fact that the ergodic theorem cannot hold for molecular dynamics simulations on accessible time scales, we probed this assumption and provided direct evidence that individual trajectories do not suffice for deriving precise, reproducible, and accurate results for protein–ligand systems. We showed on the contrary that ensembles are essential for the calculation of statistically robust results, regardless of the length of simulation for this class of systems. On comparing the protein–ligand contact frequency distributions from ten or twelve independent 10 μs trajectories, 90% or more pairs of trajectories had significantly different distributions of ligand–protein residue interactions. The principles and findings of this study are not just confined to ligand–protein systems and free energy calculations but, being based on the chaotic nature of any dynamical system which displays an equilibrium state, are more widely applicable to molecular dynamics in general and hence should be accounted for in all MD based applications regardless of the particular domain of interest.^60^

To investigate the effect of this uncertainty on the value of a one-dimensional macroscopic observable, we analyzed the same set of trajectories in order to determine ligand binding free energies and their associated statistical distributions. The specific method which we used for ligand binding free energy calculations was taken from Pan et al.^21^ In their paper, the authors reported strong correlation to FEP calculations but poor correlation to experiment, stating this poor correlation may be attributable to force field inaccuracies. In the present study, we demonstrated that separate trajectories lead to the computation of completely different results, differing by up to 7.26 kcal/mol. Our study conclusively demonstrates that binding free energies from individual simulations are inherently nonprecise and nonreproducible and do not yield chemical accuracy (±1 kcal/mol). Clearly, long time scale trajectories probe an insufficient number of microstates to effectively sample the phase space. In turn, the lack of agreement with experiment should not necessarily be attributed to force-field inaccuracies. This is a paramount example of the importance of taking aleatoric uncertainty fully into account.

In addition, by executing both the long time scale and splitting protocols, we have provided insight into the utility of adaptive sampling protocols. With respect to the length of simulations, it is clear that the merit of running a long simulation changes as a function of the time scale of events of interest. In the case of the systems studied here, no long time scale events (e.g., large-scale domain rearrangements) need to occur for ligand binding to be possible. As a result, a simple adaptive sampling protocol was able to successfully identify all of the sites identified by the long time scale protocol albeit with significantly less wall time required (1 day 7 h for adaptive sampling as compared to 43 days 2 h for 10 μs of simulation for the ADRP system).

Beyond these implications, the findings in this work also show how ensemble based computational protocols can be used to inform the process of drug discovery. For instance, with respect to ADRP, 4 binding sites that tofacitinib can bind to were identified within both the long time scale and splitting protocols. From our binding free energy analysis, we identified that tofacitinib binds to the crystallographically determined binding site with the greatest affinity out of each of ADRP the binding sites. This indicates that, in practice, tofacitinib would act as a competitive inhibitor of ADRP. Similarly, various binding sites of interest were identified for other ligand–protein complexes studied with similar conclusions made. In addition, the discovery of noncrystallographically resolved binding sites is of great interest for a future study which would aim to elucidate whether any of these binding sites can propagate allosteric effects to the substrate binding site. This would provide a novel mechanism by which to target the protein and induce antiviral effects. Finally, we compared the “direct” free energy method with ESMACS and discussed various scenarios where each method has an advantage or limitation. ESMACS is very efficient in ranking ligands based on their binding interactions with much less computational cost as compared to the direct binding affinity method. However, it is subject to the availability of a stable binding pose as the starting structure, in the absence of which long simulations do a better job. We hope that this will help others working in this domain to choose an appropriate free energy method for their purposes.

Finally, the use of ensemble methods enabled us to discover the allosteric mechanism through which the binding of a ligand at the substrate binding site of 3CLPro is affected by binding of another ligand at an experimentally known allosteric binding site. We showed that the two binding sites are connected via a β-sheet strand that causes distortion to the cavity of the substrate binding site relative to its conformation in the absence of such an allosteric effect. Such a distortion of the main binding site has a negative impact on the process of dimerization of the main protease which is essential for its activity. This explains the inhibitory effect of ligand binding at the allosteric binding site. We also discovered a novel mechanism of inhibition for a ligand hitherto only known to bind at the substrate binding site. We found that this ligand binds to an alternative binding site, blocking a key interdomain hydrogen bond leading to the rotation of domain III of the main protease and thereby preventing its dimerization and hence catalytic activity.

## Data Availability

All input structure and parameter files are available on a public github repository at https://github.com/UCL-CCS/LongTimescaleStudy.

## Supporting Information Available

Contact frequency distributions, KS statistics, p-boxes, and cumulative density functions as well as comparisons of contact frequency distributions from long simulations and splitting protocols for all systems that were not accommodated in the main text (PDF)

## References

[ref1] Berdigaliyev N.; Aljofan M. An overview of drug discovery and development. Future Medicinal Chemistry 2020, 12, 939–947. 10.4155/fmc-2019-0307. 32270704

[ref2] Bhati A. P.; Wan S.; Alfè D.; Clyde A. R.; Bode M.; Tan L.; Titov M.; Merzky A.; Turilli M.; Jha S.; Highfield R. R.; Rocchia W.; Scafuri N.; Succi S.; Kranzlmüller D.; Mathias G.; Wifling D.; Donon Y.; Di Meglio A.; Vallecorsa S.; Ma H.; Trifan A.; Ramanathan A.; Brettin T.; Partin A.; Xia F.; Duan X.; Stevens R.; Coveney P. V. Pandemic drugs at pandemic speed: infrastructure for accelerating COVID-19 drug discovery with hybrid machine learning-and physics-based simulations on high-performance computers. Interface Focus 2021, 11, 20210018 10.1098/rsfs.2021.0018. 34956592 PMC8504892

[ref3] Saadi A. A.; Alfe D.; Babuji Y.; Bhati A.; Blaiszik B.; Brettin T.; Chard K.; Chard R.; Coveney P.; Trifan A.; Brace A.; Clyde A.; Foster I.; Gibbs T.; Jha S.; Keipert K.; Kurth T.; Kranzlmüller D.; Lee H.; Li Z.; Ma H.; Merzky A.; Mathias G.; Partin A.; Yin J.; Ramanathan A.; Shah A.; Stern A.; Stevens R.; Tan L.; Titov M.; Tsaris A.; Turilli M.; Van Dam H.; Wan S.; Wifling D. IMPECCABLE: Integrated Modeling PipelinE for COVID Cure by Assessing Better LEads. Proceedings of the 50th International Conference on Parallel Processing; Association for Computing Machinery: 2021; pp 1–12.

[ref4] Buch I.; Giorgino T.; De Fabritiis G. Complete reconstruction of an enzyme-inhibitor binding process by molecular dynamics simulations. Proc. Natl. Acad. Sci. U. S. A. 2011, 108, 10184–10189. 10.1073/pnas.1103547108. 21646537 PMC3121846

[ref5] Dror R. O.; Pan A. C.; Arlow D. H.; Borhani D. W.; Maragakis P.; Shan Y.; Xu H.; Shaw D. E. Pathway and mechanism of drug binding to G-protein-coupled receptors. Proc. Natl. Acad. Sci. U. S. A. 2011, 108, 13118–13123. 10.1073/pnas.1104614108. 21778406 PMC3156183

[ref6] Shan Y.; Mysore V. P.; Leffler A. E.; Kim E. T.; Sagawa S.; Shaw D. E. How does a small molecule bind at a cryptic binding site?. bioRxiv 2021, 10.1101/2021.03.31.437917. PMC8893328 35239648

[ref7] Shan Y.; Kim E. T.; Eastwood M. P.; Dror R. O.; Seeliger M. A.; Shaw D. E. How does a drug molecule find its target binding site?. J. Am. Chem. Soc. 2011, 133, 9181–9183. 10.1021/ja202726y. 21545110 PMC3221467

[ref8] Silva D.-A.; Bowman G. R.; Sosa-Peinado A.; Huang X. A role for both conformational selection and induced fit in ligand binding by the LAO protein. PLoS Computational Biology 2011, 7, e1002054 10.1371/journal.pcbi.1002054. 21637799 PMC3102756

[ref9] Gu S.; Silva D.-A.; Meng L.; Yue A.; Huang X. Quantitatively Characterizing the Ligand Binding Mechanisms of Choline Binding Protein Using Markov State Model Analysis. PLOS Computational Biology 2014, 10, e1003767 10.1371/journal.pcbi.1003767. 25101697 PMC4125059

[ref10] Ahalawat N.; Mondal J. Mapping the Substrate Recognition Pathway in Cytochrome P450. J. Am. Chem. Soc. 2018, 140, 17743–17752. 10.1021/jacs.8b10840. 30479124

[ref11] Betz R. M.; Dror R. O. How Effectively Can Adaptive Sampling Methods Capture Spontaneous Ligand Binding?. J. Chem. Theory Comput. 2019, 15, 2053–2063. 10.1021/acs.jctc.8b00913. 30645108 PMC6795214

[ref12] Shaw D. E.; Grossman J.; Bank J. A.; Batson B.; Butts J. A.; Chao J. C.; Deneroff M. M.; Dror R. O.; Even A.; Fenton C. H., Anton 2: raising the bar for performance and programmability in a special-purpose molecular dynamics supercomputer. SC’14: Proceedings of the International Conference for High Performance Computing, Networking, Storage and Analysis; Association for Computing Machinery: 2014; pp 41–53.

[ref13] Shaw D. E.; Adams P. J.; Azaria A.; Bank J. A.; Batson B.; Bell A.; Bergdorf M.; Bhatt J.; Butts J. A.; Correia T., Anton 3: twenty microseconds of molecular dynamics simulation before lunch. SC’21: Proceedings of the International Conference for High Performance Computing, Networking, Storage and Analysis; Association for Computing Machinery: 2021; pp 1–11.

[ref14] Bieniek M. K.; Bhati A. P.; Wan S.; Coveney P. V. TIES 20: Relative Binding Free Energy with a Flexible Superimposition Algorithm and Partial Ring Morphing. J. Chem. Theory Comput. 2021, 17, 1250–1265. 10.1021/acs.jctc.0c01179. 33486956 PMC7876800

[ref15] Coveney P. V.; Wan S. On the calculation of equilibrium thermodynamic properties from molecular dynamics. Phys. Chem. Chem. Phys. 2016, 18, 30236–30240. 10.1039/C6CP02349E. 27165501

[ref16] Bhati A. P.; Wan S.; Wright D. W.; Coveney P. V. Rapid, accurate, precise, and reliable relative free energy prediction using ensemble based thermodynamic integration. J. Chem. Theory Comput. 2017, 13, 210–222. 10.1021/acs.jctc.6b00979. 27997169

[ref17] Bhati A. P.; Wan S.; Hu Y.; Sherborne B.; Coveney P. V. Uncertainty Quantification in Alchemical Free Energy Methods. J. Chem. Theory Comput. 2018, 14, 2867–2880. 10.1021/acs.jctc.7b01143. 29678106 PMC6095638

[ref18] Vassaux M.; Wan S.; Edeling W.; Coveney P. V. Ensembles Are Required to Handle Aleatoric and Parametric Uncertainty in Molecular Dynamics Simulation. J. Chem. Theory Comput. 2021, 17, 5187–5197. 10.1021/acs.jctc.1c00526. 34280310 PMC8389531

[ref19] Pande V. S.; Beauchamp K.; Bowman G. R. Everything you wanted to know about Markov State Models but were afraid to ask. Methods 2010, 52, 99–105. Protein Folding 10.1016/j.ymeth.2010.06.002. 20570730 PMC2933958

[ref20] Bowman G. R.; Huang X.; Pande V. S. Using generalized ensemble simulations and Markov state models to identify conformational states. Methods 2009, 49, 197–201. RNA Dynamics 10.1016/j.ymeth.2009.04.013. 19410002 PMC2753735

[ref21] Pan A. C.; Xu H.; Palpant T.; Shaw D. E. Quantitative characterization of the binding and unbinding of millimolar drug fragments with molecular dynamics simulations. J. Chem. Theory Comput. 2017, 13, 3372–3377. 10.1021/acs.jctc.7b00172. 28582625

[ref22] Plattner N.; Noé F. Protein conformational plasticity and complex ligand-binding kinetics explored by atomistic simulations and Markov models. Nat. Commun. 2015, 6, 7653 10.1038/ncomms8653. 26134632 PMC4506540

[ref23] Paul F.; Wehmeyer C.; Abualrous E. T.; Wu H.; Crabtree M. D.; Schöneberg J.; Clarke J.; Freund C.; Weikl T. R.; Noé F. Protein-peptide association kinetics beyond the seconds timescale from atomistic simulations. Nat. Commun. 2017, 8, 1095 10.1038/s41467-017-01163-6. 29062047 PMC5653669

[ref24] Dror R. O.; Green H. F.; Valant C.; Borhani D. W.; Valcourt J. R.; Pan A. C.; Arlow D. H.; Canals M.; Lane J. R.; Rahmani R.; Baell J. B.; Sexton P. M.; Christopoulos A.; Shaw D. E. Structural basis for modulation of a G-protein-coupled receptor by allosteric drugs. Nature 2013, 503, 295–299. 10.1038/nature12595. 24121438

[ref25] Doerr S.; De Fabritiis G. On-the-Fly Learning and Sampling of Ligand Binding by High-Throughput Molecular Simulations. J. Chem. Theory Comput. 2014, 10, 2064–2069. 10.1021/ct400919u. 26580533

[ref26] Kohlhoff K. J.; Shukla D.; Lawrenz M.; Bowman G. R.; Konerding D. E.; Belov D.; Altman R. B.; Pande V. S. Cloud-based simulations on Google Exacycle reveal ligand modulation of GPCR activation pathways. Nat. Chem. 2014, 6, 15–21. 10.1038/nchem.1821. 24345941 PMC3923464

[ref27] Bowman G. R.; Beauchamp K. A.; Boxer G.; Pande V. S. Progress and challenges in the automated construction of Markov state models for full protein systems. J. Chem. Phys. 2009, 131, 124101 10.1063/1.3216567. 19791846 PMC2766407

[ref28] Noé F.; Fischer S. Transition networks for modeling the kinetics of conformational change in macromolecules. Curr. Opin. Struct. Biol. 2008, 18, 154–162. Theory and simulation/Macromolecular assemblages 10.1016/j.sbi.2008.01.008. 18378442

[ref29] Voter A. F. Parallel replica method for dynamics of infrequent events. Phys. Rev. B 1998, 57, R13985–R13988. 10.1103/PhysRevB.57.R13985.

[ref30] Shirts M. R.; Pande V. S. Mathematical Analysis of Coupled Parallel Simulations. Phys. Rev. Lett. 2001, 86, 4983–4987. 10.1103/PhysRevLett.86.4983. 11384401

[ref31] Zagrovic B.; Sorin E. J.; Pande V. β-hairpin folding simulations in atomistic detail using an implicit solvent model11Edited by F. Cohen. J. Mol. Biol. 2001, 313, 151–169. 10.1006/jmbi.2001.5033. 11601853

[ref32] Snow C. D.; Nguyen H.; Pande V. S.; Gruebele M. Absolute comparison of simulated and experimental protein-folding dynamics. Nature 2002, 420, 102–106. 10.1038/nature01160. 12422224

[ref33] Swope W. C.; Pitera J. W.; Suits F. Describing Protein Folding Kinetics by Molecular Dynamics Simulations. 1. Theory. J. Phys. Chem. B 2004, 108, 6571–6581. 10.1021/jp037421y.

[ref34] Ensign D. L.; Kasson P. M.; Pande V. S. Heterogeneity Even at the Speed Limit of Folding: Large-scale Molecular Dynamics Study of a Fast-folding Variant of the Villin Headpiece. J. Mol. Biol. 2007, 374, 806–816. 10.1016/j.jmb.2007.09.069. 17950314 PMC3689540

[ref35] Jayachandran G.; Vishal V.; Pande V. S. Using massively parallel simulation and Markovian models to study protein folding: Examining the dynamics of the villin headpiece. J. Chem. Phys. 2006, 124, 164902 10.1063/1.2186317. 16674165

[ref36] Chodera J. D.; Swope W. C.; Pitera J. W.; Dill K. A. Long-Time Protein Folding Dynamics from Short-Time Molecular Dynamics Simulations. Multiscale Modeling & Simulation 2006, 5, 1214–1226. 10.1137/06065146X.

[ref37] Singhal N.; Pande V. S. Error analysis and efficient sampling in Markovian state models for molecular dynamics. J. Chem. Phys. 2005, 123, 204909 10.1063/1.2116947. 16351319

[ref38] Chodera J. D.; Singhal N.; Pande V. S.; Dill K. A.; Swope W. C. Automatic discovery of metastable states for the construction of Markov models of macromolecular conformational dynamics. J. Chem. Phys. 2007, 126, 155101 10.1063/1.2714538. 17461665

[ref39] Huber G.; Kim S. Weighted-ensemble Brownian dynamics simulations for protein association reactions. Biophys. J. 1996, 70, 97–110. 10.1016/S0006-3495(96)79552-8. 8770190 PMC1224912

[ref40] Zuckerman D. M.; Chong L. T. Weighted Ensemble Simulation: Review of Methodology, Applications, and Software. Annual Review of Biophysics 2017, 46, 43–57. 10.1146/annurev-biophys-070816-033834. PMC5896317 28301772

[ref41] Dickson A. Mapping the Ligand Binding Landscape. Biophys. J. 2018, 115, 1707–1719. 10.1016/j.bpj.2018.09.021. 30327139 PMC6224774

[ref42] Bhatt D.; Zhang B. W.; Zuckerman D. M. Steady-state simulations using weighted ensemble path sampling. J. Chem. Phys. 2010, 133, 014110 10.1063/1.3456985. 20614962 PMC2912933

[ref43] Bhatt D.; Bahar I. An adaptive weighted ensemble procedure for efficient computation of free energies and first passage rates. J. Chem. Phys. 2012, 137, 104101 10.1063/1.4748278. 22979844 PMC3460967

[ref44] Adelman J. L.; Grabe M. Simulating rare events using a weighted ensemble-based string method. J. Chem. Phys. 2013, 138, 044105 10.1063/1.4773892. 23387566 PMC3568092

[ref45] Suárez E.; Lettieri S.; Zwier M. C.; Stringer C. A.; Subramanian S. R.; Chong L. T.; Zuckerman D. M. Simultaneous Computation of Dynamical and Equilibrium Information Using a Weighted Ensemble of Trajectories. J. Chem. Theory Comput. 2014, 10, 2658–2667. 10.1021/ct401065r. 25246856 PMC4168800

[ref46] Zwier M. C.; Adelman J. L.; Kaus J. W.; Pratt A. J.; Wong K. F.; Rego N. B.; Suárez E.; Lettieri S.; Wang D. W.; Grabe M.; Zuckerman D. M.; Chong L. T. WESTPA: An Interoperable, Highly Scalable Software Package for Weighted Ensemble Simulation and Analysis. J. Chem. Theory Comput. 2015, 11, 800–809. 10.1021/ct5010615. 26392815 PMC4573570

[ref47] Dickson A.; Brooks C. L. WExplore: Hierarchical Exploration of High-Dimensional Spaces Using the Weighted Ensemble Algorithm. J. Phys. Chem. B 2014, 118, 3532–3542. 10.1021/jp411479c. 24490961 PMC4404516

[ref48] Abdul-Wahid B.; Feng H.; Rajan D.; Costaouec R.; Darve E.; Thain D.; Izaguirre J. A. AWE-WQ: Fast-Forwarding Molecular Dynamics Using the Accelerated Weighted Ensemble. J. Chem. Inf. Model. 2014, 54, 3033–3043. 10.1021/ci500321g. 25207854 PMC4210180

[ref49] Glasserman P.; Heidelberger P.; Shahabuddin P.; Zajic T. Splitting for rare event simulation: analysis of simple cases. Proceedings of the 28th Conferenence on Winter Simulation 1996, 302–308. 10.1145/256562.256635.

[ref50] Glasserman P.; Heidelberger P.; Shahabuddin P.; Zajic T. Multilevel Splitting for Estimating Rare Event Probabilities. Operations Research 1999, 47, 585–600. 10.1287/opre.47.4.585.

[ref51] Cérou F.; Guyader A. Adaptive Multilevel Splitting for Rare Event Analysis. Stochastic Analysis and Applications 2007, 25, 417–443. 10.1080/07362990601139628.

[ref52] Teo I.; Mayne C. G.; Schulten K.; Lelièvre T. Adaptive Multilevel Splitting Method for Molecular Dynamics Calculation of Benzamidine-Trypsin Dissociation Time. J. Chem. Theory Comput. 2016, 12, 2983–2989. 10.1021/acs.jctc.6b00277. 27159059 PMC5724379

[ref53] Cérou F.; Guyader A.; Rousset M. Adaptive multilevel splitting: Historical perspective and recent results. Chaos: An Interdisciplinary Journal of Nonlinear Science 2019, 29, 043108 10.1063/1.5082247. 31042959

[ref54] Fukunishi H.; Watanabe O.; Takada S. On the Hamiltonian replica exchange method for efficient sampling of biomolecular systems: Application to protein structure prediction. J. Chem. Phys. 2002, 116, 9058–9067. 10.1063/1.1472510.

[ref55] Wang L.; Friesner R. A.; Berne B. J. Replica Exchange with Solute Scaling: A More Efficient Version of Replica Exchange with Solute Tempering (REST2). J. Phys. Chem. B 2011, 115, 9431–9438. 10.1021/jp204407d. 21714551 PMC3172817

[ref56] Bhati A. P.; Wan S.; Coveney P. V. Ensemble-Based Replica Exchange Alchemical Free Energy Methods: The Effect of Protein Mutations on Inhibitor Binding. J. Chem. Theory Comput. 2019, 15, 1265–1277. 10.1021/acs.jctc.8b01118. 30592603 PMC6447239

[ref57] Michalska K.; Kim Y.; Jedrzejczak R.; Maltseva N. I.; Stols L.; Endres M.; Joachimiak A. Crystal structures of SARS-CoV-2 ADP-ribose phosphatase: from the apo form to ligand complexes. IUCrJ. 2020, 7, 814–824. 10.1107/S2052252520009653. 32939273 PMC7467174

[ref58] Fu Z.; Huang B.; Tang J.; Liu S.; Liu M.; Ye Y.; Liu Z.; Xiong Y.; Zhu W.; Cao D.; Li J.; Niu X.; Zhou H.; Zhao Y. J.; Zhang G.; Huang H. The complex structure of GRL0617 and SARS-CoV-2 PLpro reveals a hot spot for antiviral drug discovery. Nat. Commun. 2021, 12, 488 10.1038/s41467-020-20718-8. 33473130 PMC7817691

[ref59] Su H.-x.; Yao S.; Zhao W.-f.; Li M.-j.; Liu J.; Shang W.-j.; Xie H.; Ke C.-q.; Hu H.-c.; Gao M.-n.; Yu K.-q.; Liu H.; Shen J.-s.; Tang W.; Zhang L.-k.; Xiao G.-f.; Ni L.; Wang D.-w.; Zuo J.-p.; Jiang H.-l.; Bai F.; Wu Y.; Ye Y.; Xu Y.-c. Anti-SARS-CoV-2 activities in vitro of Shuanghuanglian preparations and bioactive ingredients. Acta Pharmacologica Sinica 2020, 41, 1167–1177. 10.1038/s41401-020-0483-6. 32737471 PMC7393338

[ref60] Wan S.; Sinclair R. C.; Coveney P. V. Uncertainty quantification in classical molecular dynamics. Philosophical Transactions of the Royal Society A 2021, 379, 20200082 10.1098/rsta.2020.0082. PMC8059622 33775140

[ref61] Wan S.; Bhati A. P.; Zasada S. J.; Coveney P. V. Rapid, accurate, precise and reproducible ligand–protein binding free energy prediction. J. R. Soc. Interface Focus 2020, 10, 20200007 10.1098/rsfs.2020.0007. PMC7653346 33178418

[ref62] Wan S.; Bhati A. P.; Wade A. D.; Alfè D.; Coveney P. V. Thermodynamic and structural insights into the repurposing of drugs that bind to SARS-CoV-2 main protease. Mol. Syst. Des. Eng. 2022, 7, 123–131. 10.1039/D1ME00124H. 35223088 PMC8820189

[ref63] Günther S.; Meents A.; Reinke P. Y.; Fernández-García Y.; Lieske J.; Lane T. J.; Ginn H. M.; Koua F. H.; Ehrt C.; Ewert W.; Oberthuer D.; et al. X-ray screening identifies active site and allosteric inhibitors of SARS-CoV-2 main protease. Science 2021, 372, 642–646. 10.1126/science.abf7945. 33811162 PMC8224385

[ref64] Cohen S. B.; Tanaka Y.; Mariette X.; Curtis J. R.; Lee E. B.; Nash P.; Winthrop K. L.; Charles-Schoeman C.; Thirunavukkarasu K.; DeMasi R.; Geier J.; Kwok K.; Wang L.; Riese R.; Wollenhaupt J. Long-term safety of tofacitinib for the treatment of rheumatoid arthritis up to 8.5 years: integrated analysis of data from the global clinical trials. Annals of the Rheumatic Diseases 2017, 76, 1253–1262. 10.1136/annrheumdis-2016-210457. 28143815 PMC5530353

[ref65] Kucharz J. E.; Stajszczyk M.; Kotulska-Kucharz A.; Batko B.; Brzosko M.; Jeka S.; Leszczyński P.; Majdan M.; Olesińska M.; Samborski W.; Wiland P. Tofacitinib in the treatment of patients with rheumatoid arthritis: position statement of experts of the Polish Society for Rheumatology. Reumatologia/Rheumatology 2018, 56, 203–211. 10.5114/reum.2018.77971. 30237624 PMC6142023

[ref66] Wan S.; Bhati A. P.; Zasada S. J.; Wall I.; Green D.; Bamborough P.; Coveney P. V. Rapid and Reliable Binding Affinity Prediction of Bromodomain Inhibitors: A Computational Study. J. Chem. Theory Comput. 2017, 13, 784–795. 10.1021/acs.jctc.6b00794. 28005370 PMC5312866

[ref67] Shapovalov M. V.; Dunbrack R. L. Jr A smoothed backbone-dependent rotamer library for proteins derived from adaptive kernel density estimates and regressions. Structure 2011, 19, 844–858. 10.1016/j.str.2011.03.019. 21645855 PMC3118414

[ref68] Pettersen E. F.; Goddard T. D.; Huang C. C.; Couch G. S.; Greenblatt D. M.; Meng E. C.; Ferrin T. E. UCSF Chimera—a visualization system for exploratory research and analysis. J. Comput. Chem. 2004, 25, 1605–1612. 10.1002/jcc.20084. 15264254

[ref69] Kalé L.; Skeel R.; Bhandarkar M.; Brunner R.; Gursoy A.; Krawetz N.; Phillips J.; Shinozaki A.; Varadarajan K.; Schulten K. NAMD2: greater scalability for parallel molecular dynamics. J. Comput. Phys. 1999, 151, 283–312. 10.1006/jcph.1999.6201.

[ref70] Shan Y.; Mysore V. P.; Leffler A. E.; Kim E. T.; Sagawa S.; Shaw D. E. How does a small molecule bind at a cryptic binding site?. PLOS Computational Biology 2022, 18, e1009817 10.1371/journal.pcbi.1009817. 35239648 PMC8893328

[ref71] Robustelli P.; Ibanez-de Opakua A.; Campbell-Bezat C.; Giordanetto F.; Becker S.; Zweckstetter M.; Pan A. C.; Shaw D. E. Molecular Basis of Small-Molecule Binding to α-Synuclein. J. Am. Chem. Soc. 2022, 144, 2501–2510. 10.1021/jacs.1c07591. 35130691 PMC8855421

[ref72] Phillips J. C.; Hardy D. J.; Maia J. D.; Stone J. E.; Ribeiro J. V.; Bernardi R. C.; Buch R.; Fiorin G.; Hénin J.; Jiang W.; McGreevy R.; Melo M. C. R.; Radak B. K.; Skeel R. D.; Singharoy A.; Wang Y.; Roux B.; Aksimentiev A.; Luthey-Schulten Z.; Kalé L. V.; Schulten K.; Chipot C.; Tajkhorshid E. Scalable molecular dynamics on CPU and GPU architectures with NAMD. J. Chem. Phys. 2020, 153, 044130 10.1063/5.0014475. 32752662 PMC7395834

[ref73] Eastman P.; Swails J.; Chodera J. D.; McGibbon R. T.; Zhao Y.; Beauchamp K. A.; Wang L.-P.; Simmonett A. C.; Harrigan M. P.; Stern C. D.; Wiewiora R. P.; Brooks B. R.; Pande V. S. OpenMM 7: Rapid development of high performance algorithms for molecular dynamics. PLoS Computational Biology 2017, 13, e1005659 10.1371/journal.pcbi.1005659. 28746339 PMC5549999

[ref74] Venkatakrishnan A.; Fonseca R.; Ma A. K.; Hollingsworth S. A.; Chemparathy A.; Hilger D.; Kooistra A. J.; Ahmari R.; Babu M. M.; Kobilka B. K.; Dror R. O. Uncovering patterns of atomic interactions in static and dynamic structures of proteins. bioRxiv 2019, 840694 10.1101/840694.

[ref75] De Jong D. H.; Schäfer L. V.; De Vries A. H.; Marrink S. J.; Berendsen H. J.; Grubmüller H. Determining equilibrium constants for dimerization reactions from molecular dynamics simulations. J. Comput. Chem. 2011, 32, 1919–1928. 10.1002/jcc.21776. 21469160

[ref76] Kollman P. A.; Massova I.; Reyes C.; Kuhn B.; Huo S.; Chong L.; Lee M.; Lee T.; Duan Y.; Wang W.; Donini O.; Cieplak P.; Srinivasan J.; Case D. A.; Cheatham T. E. Calculating Structures and Free Energies of Complex Molecules: Combining Molecular Mechanics and Continuum Models. Acc. Chem. Res. 2000, 33, 889–897. 10.1021/ar000033j. 11123888

[ref77] Case D.A.; Belfon K.; Ben-Shalom I. Y.; Brozell S.R.; Cerutti D.S.; Cheatham T.E. III; Cruzeiro V.W.D.; Darden T.A.; Duke R.E.; Giambasu G.; Gilson M.K.; Gohlke H.; Goetz A.W.; Harris R.; Izadi S.; Izmailov S.A.; Kasavajhala K.; Kovalenko A.; Krasny R.; Kurtzman T.; Lee T.S.; LeGrand S.; Li P.; Lin C.; Liu J.; Luchko T.; Luo R.; Man V.; Merz K.M.; Miao Y.; Mikhailovskii O.; Monard G.; Nguyen H.; Onufriev A.; Pan F.; Pantano S.; Qi R.; Roe D.R.; Roitberg A.; Sagui C.; Schott-Verdugo S.; Shen J.; Simmerling C.L.; Skrynnikov N. R.; Smith J.; Swails J.; Walker R.C.; Wang J.; Wilson L.; Wolf R.M.; Wu X.; Xiong Y.; Xue Y.; York D.M.; Kollman P.A. AMBER 2020; University of California, San Francisco, 2020.

[ref78] Virtanen P.; Gommers R.; Oliphant T. E.; Haberland M.; Reddy T.; Cournapeau D.; Burovski E.; Peterson P.; Weckesser W.; Bright J.; van der Walt S. J.; Brett M.; Wilson J.; Millman K. J.; Mayorov N.; Nelson A. R. J.; Jones E.; Kern R.; Larson E.; Carey C. J.; Polat İ.; Feng Y.; Moore E. W.; VanderPlas J.; Laxalde D.; Perktold J.; Cimrman R.; Henriksen I.; Quintero E. A.; Harris C. R.; Archibald A. M.; Ribeiro A. H.; Pedregosa F.; van Mulbregt P.; SciPy 1.0: Fundamental Algorithms for Scientific Computing in Python. Nat. Methods 2020, 17, 261–272. 10.1038/s41592-019-0686-2. 32015543 PMC7056644

[ref79] Oberkampf W. L.; Roy C. J. Verification and Validation in Scientific Computing; Cambridge University Press, 2010.

[ref80] Bhati A. P.; Coveney P. V. Large Scale Study of Ligand–Protein Relative Binding Free Energy Calculations: Actionable Predictions from Statistically Robust Protocols. J. Chem. Theory Comput. 2022, 18, 2687–2702. 10.1021/acs.jctc.1c01288. 35293737 PMC9009079

[ref81] Wade A. D.; Bhati A. P.; Wan S.; Coveney P. V. Alchemical Free Energy Estimators and Molecular Dynamics Engines: Accuracy, Precision, and Reproducibility. J. Chem. Theory Comput. 2022, 18, 3972–3987. 10.1021/acs.jctc.2c00114. 35609233 PMC9202356

[ref82] Alzyoud L.; Ghattas M. A.; Atatreh N. Allosteric Binding Sites of the SARS-CoV-2 Main Protease: Potential Targets for Broad-Spectrum Anti-Coronavirus Agents. Drug Design, Development and Therapy 2022, 16, 2463–2478. 10.2147/DDDT.S370574. 35941927 PMC9356625

[ref83] Zhang L.; Lin D.; Sun X.; Curth U.; Drosten C.; Sauerhering L.; Becker S.; Rox K.; Hilgenfeld R. Crystal structure of SARS-CoV-2 main protease provides a basis for design of improved α-ketoamide inhibitors. Science 2020, 368, 409–412. 10.1126/science.abb3405. 32198291 PMC7164518

[ref84] Shi J.; Sivaraman J.; Song J. Mechanism for Controlling the Dimer-Monomer Switch and Coupling Dimerization to Catalysis of the Severe Acute Respiratory Syndrome Coronavirus 3C-Like Protease. Journal of Virology 2008, 82, 4620–4629. 10.1128/JVI.02680-07. 18305031 PMC2293028

[ref85] Frenkel D.; Smit B. Understanding Molecular Simulation: From Algorithms to Applications, 2nd ed.; Elsevier, 2001; pp 15–17.

[ref86] Haile J. Molecular Dynamics Simulation: Elementary Methods; Wiley, 1997; pp 15–16.

[ref87] Skeel R. D. What Makes Molecular Dynamics Work?. SIAM Journal on Scientific Computing 2009, 31, 1363–1378. 10.1137/070683660. 20084278 PMC2800798

