# Supplementary material for: Long Time Scale Ensemble Methods in Molecular Dynamics: Ligand–Protein Interactions and Allostery in SARS-CoV-2 Targets
Source: J Chem Theory Comput. 2023 May 29;19(11):3359–78. doi: 10.1021/acs.jctc.3c00020 (PMC10241325; doi:10.1021/acs.jctc.3c00020)
Supplement: Supplementary file 1 — ct3c00020_si_001.pdf [file ct3c00020_si_001.pdf]

Supporting Information: Long timescale ensemble methods in molecular dynamics: Ligand-protein interactions and allostery in SARS-CoV-2 targets

Agastya P. Bhati,† Art Hoti,† Andrew Potterton,‡ Mateusz K. Bieniek,† and Peter V. Coveney*,†,¶,§

†*Centre for Computational Science, Department of Chemistry, University College London, London, United Kingdom*

‡*BenevolentAI, London, W1T 5HD, United Kingdom*

¶*Computational Science Laboratory, Institute for Informatics, Faculty of Science, University of Amsterdam, Amsterdam,The Netherlands*

§*Advanced Research Computing Centre, University College London, London, United Kingdom*

E-mail: p.v.coveney@ucl.ac.uk

Phone: +44 (0)20 7679 4560

Here, we include the supporting information for this study. Figures displaying contact frequency distributions, KS statistics, p-boxes and cumulative density functions as well as comparisons of contact frequency distributions from long simulations and splitting protocols have been included for all those systems that we were unable to accommodate in the main text. We have studied 5 different protein-ligand systems and generated a large amount of data.

APB and AH contributed equally to this work

However, the results and implications obtained are very similar for all systems. Therefore, to avoid making the main text very lengthy, we only included a representative figure in the main text (only one system in most cases) displaying the general findings and similar/identical figures for all other systems have been included in this document.

Free Energy Methods: Direct versus ESMACS

When performing "long" simulations, a large number of binding poses are observed and it is hard to identify the most stable one in the absence of available experimental information. In such cases, ESMACS-s is not so useful as resultant ΔG values may vary substantially. As an example, we randomly picked out two different binding poses of 93J sampled within the long trajectories of 3CLPro-93J system at three binding sites (A, B and H1) and performed ESMACS-s calculations using each of them as the starting structures. The differences in $\Delta G_{ESMACS-s}$ values obtained starting from the two different binding poses at each 93J binding site are 11.44 kcal/mol, 4.93 kcal/mol and 6.61 kcal/mol respectively which are quite large and can result in very different rankings.

Table S1: Free energies obtained using different protocols. "ESMACS-s" and "ESMACS-l" correspond to free energies obtained using the standard ESMACS protocol (initiated from a chosen conformation) and those using bound conformations extracted from "long" trajectories, respectively. Error bars are included in brackets and denote the standard errors across all replicas that sample a given binding site. All values are in kcal/mol.

Binding site	Direct	ESMACS-s	ESMACS-l
A	-2.45(0.35)	-12.94(0.17)	-16.21(0.47)
B	-2.40(0.17)	-17.11(0.10)	-20.34(0.45)
C	-4.03(0.20)	-27.32(0.16)	-36.97(1.02)
D	-2.08(0.08)	-17.97(0.43)	-19.18(0.71)

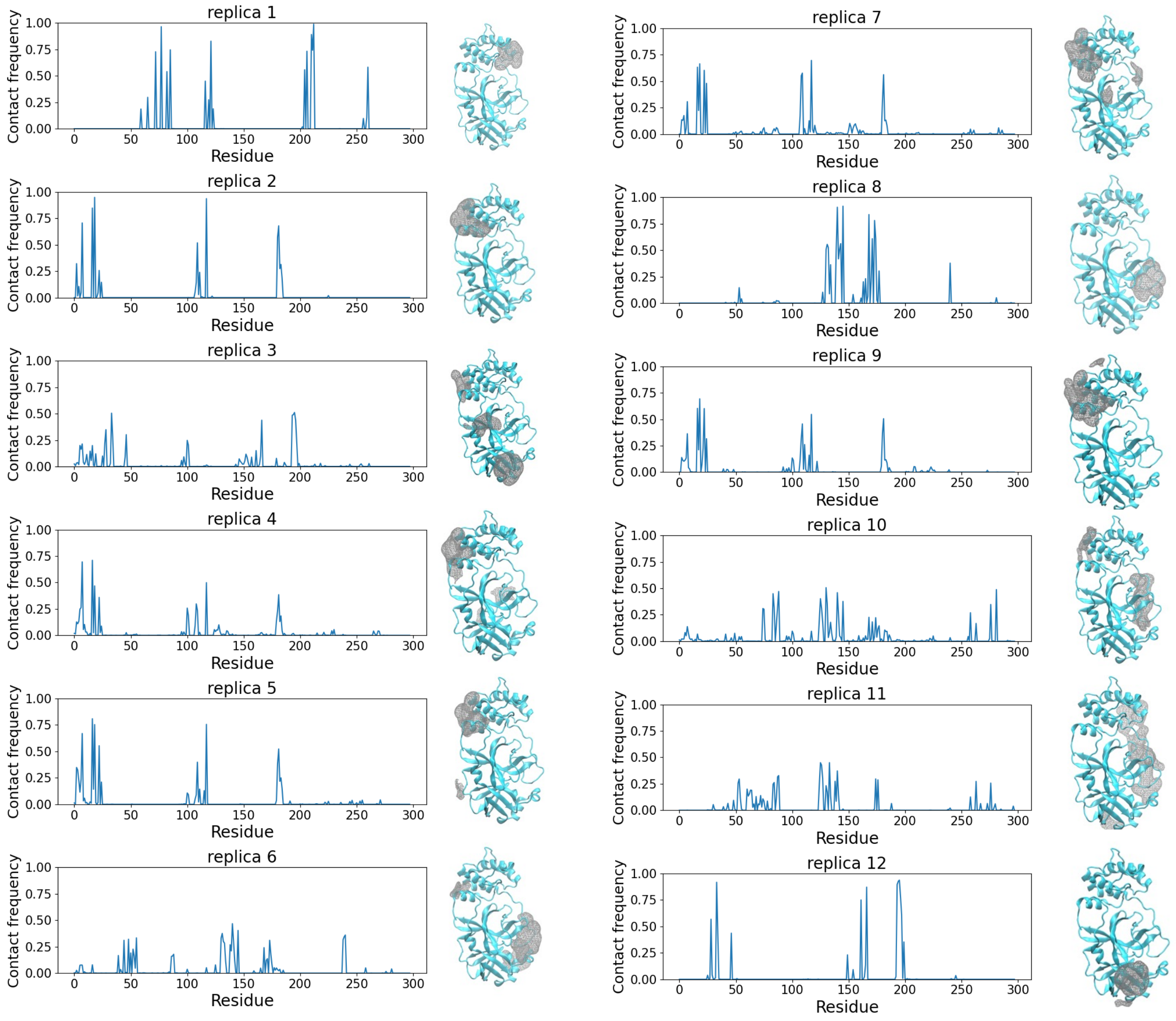

Figure S1: Ligand-protein residue contact frequency distribution plots for each “long” replica are shown adjacent to their respective ligand occupancy maps for the 3CLPro-93J system. The ligand-residue contact frequencies correspond to the fraction of frames in which a hydrophobic contact is formed between the ligand and a given protein residue. Occupancy maps of the ligand around the protein represent the isovalue surfaces (wireframe representation) rendered at a fractional occupancy of 0.03 across all frames of the simulation trajectory. In other words, they represent volumes of the simulation box where the ligand is likely to be found with 97% probability, that is in 97% of all trajectory frames.

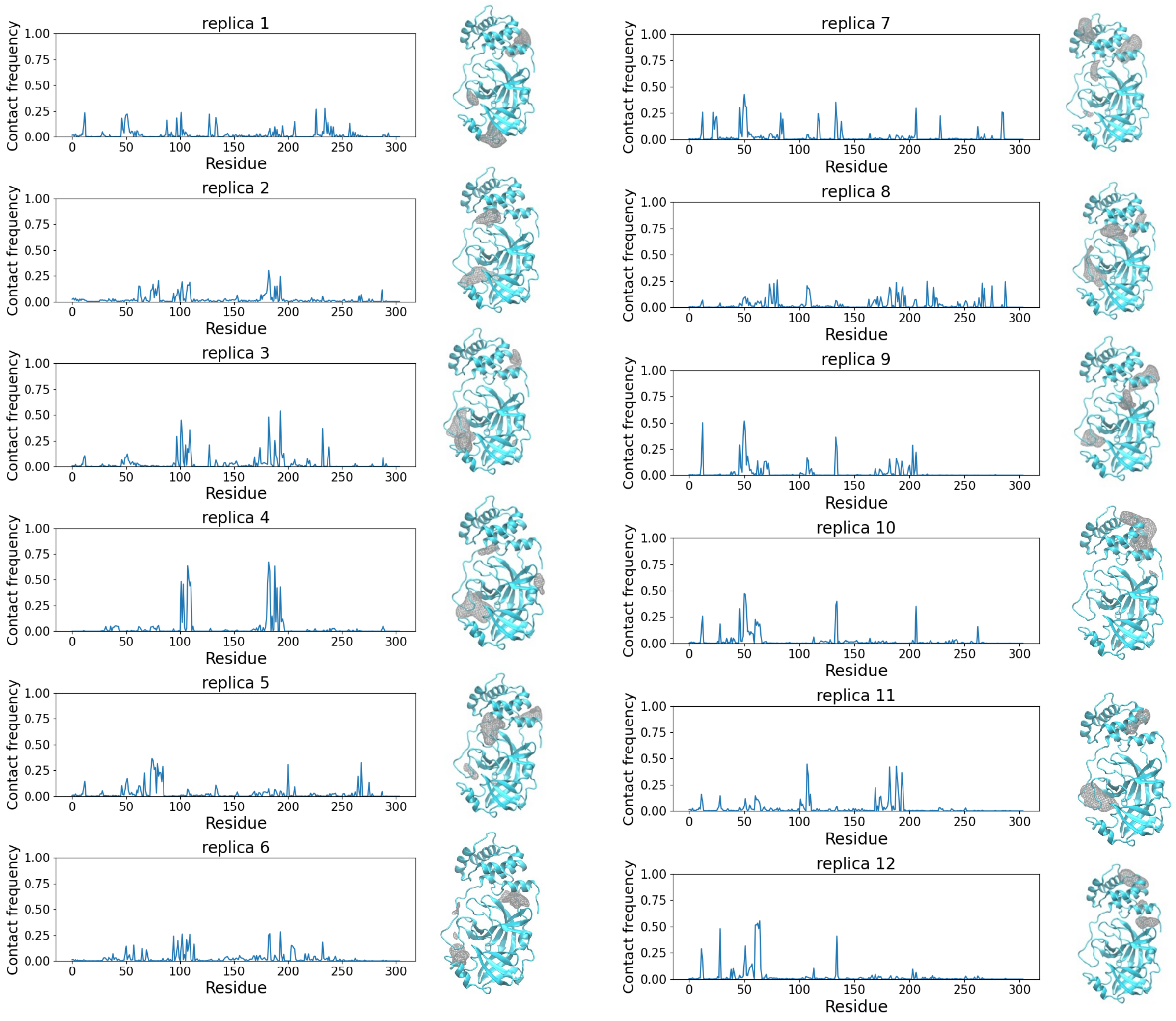

Figure S2: Ligand-protein residue contact frequency distribution plots for each “long” replica are shown adjacent to their respective ligand occupancy maps for the 3CLPro-RQN system. The ligand-residue contact frequencies correspond to the fraction of frames in which a hydrophobic contact is formed between the ligand and a given protein residue. Occupancy maps of the ligand around the protein represent the isovalue surfaces (wireframe representation) rendered at a fractional occupancy of 0.03 across all frames of the simulation trajectory. In other words, they represent volumes of the simulation box where the ligand is likely to be found with 97% probability, that is in 97% of all trajectory frames.

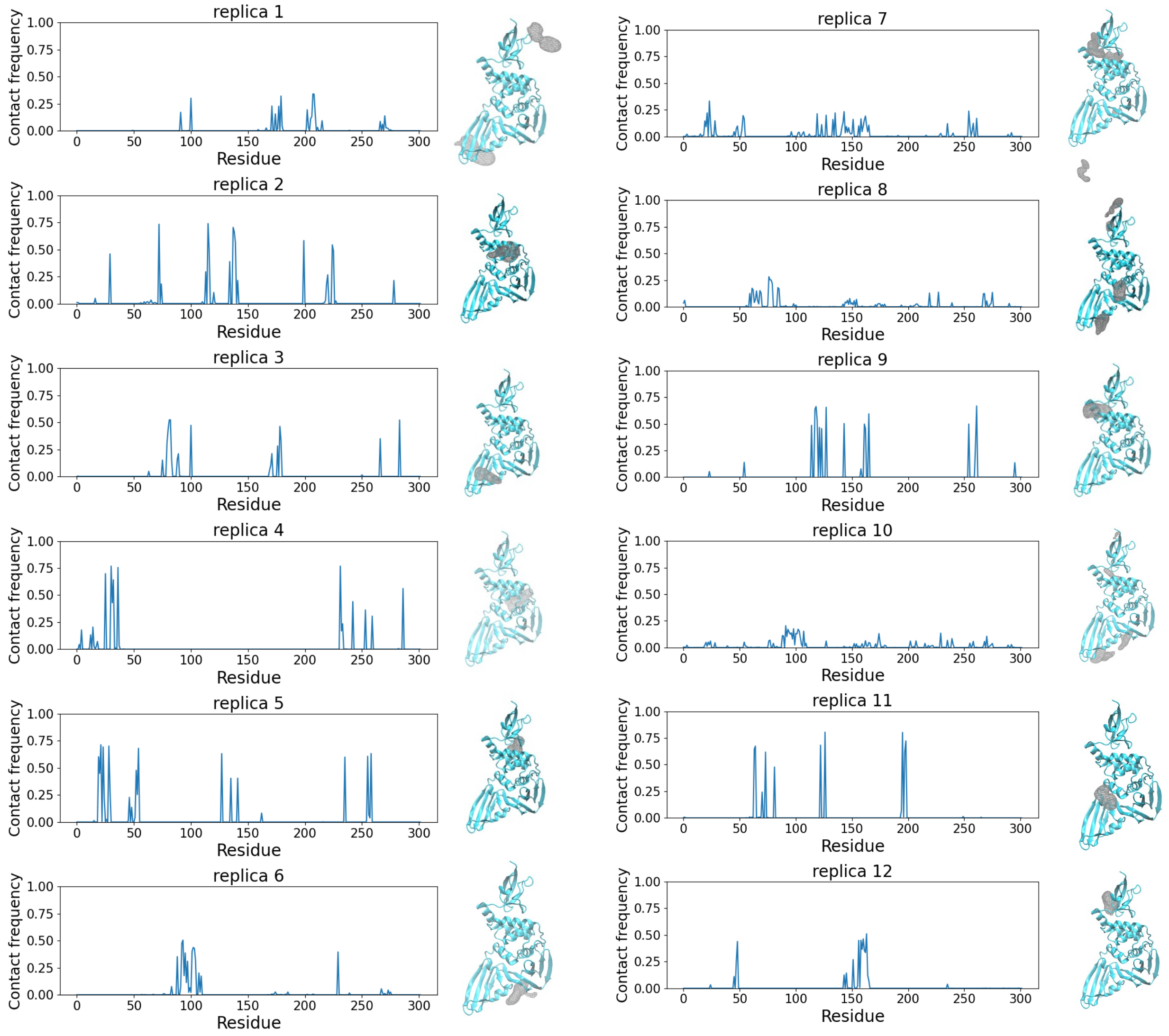

Figure S3: Ligand-protein residue contact frequency distribution plots for each "long" replica are shown adjacent to their respective ligand occupancy maps for the PLPro-GRL system. The ligand-residue contact frequencies correspond to the fraction of frames in which a hydrophobic contact is formed between the ligand and a given protein residue. Occupancy maps of the ligand around the protein represent the isovalue surfaces (wireframe representation) rendered at a fractional occupancy of 0.03 across all frames of the simulation trajectory. In other words, they represent volumes of the simulation box where the ligand is likely to be found with 97% probability, that is in 97% of all trajectory frames.

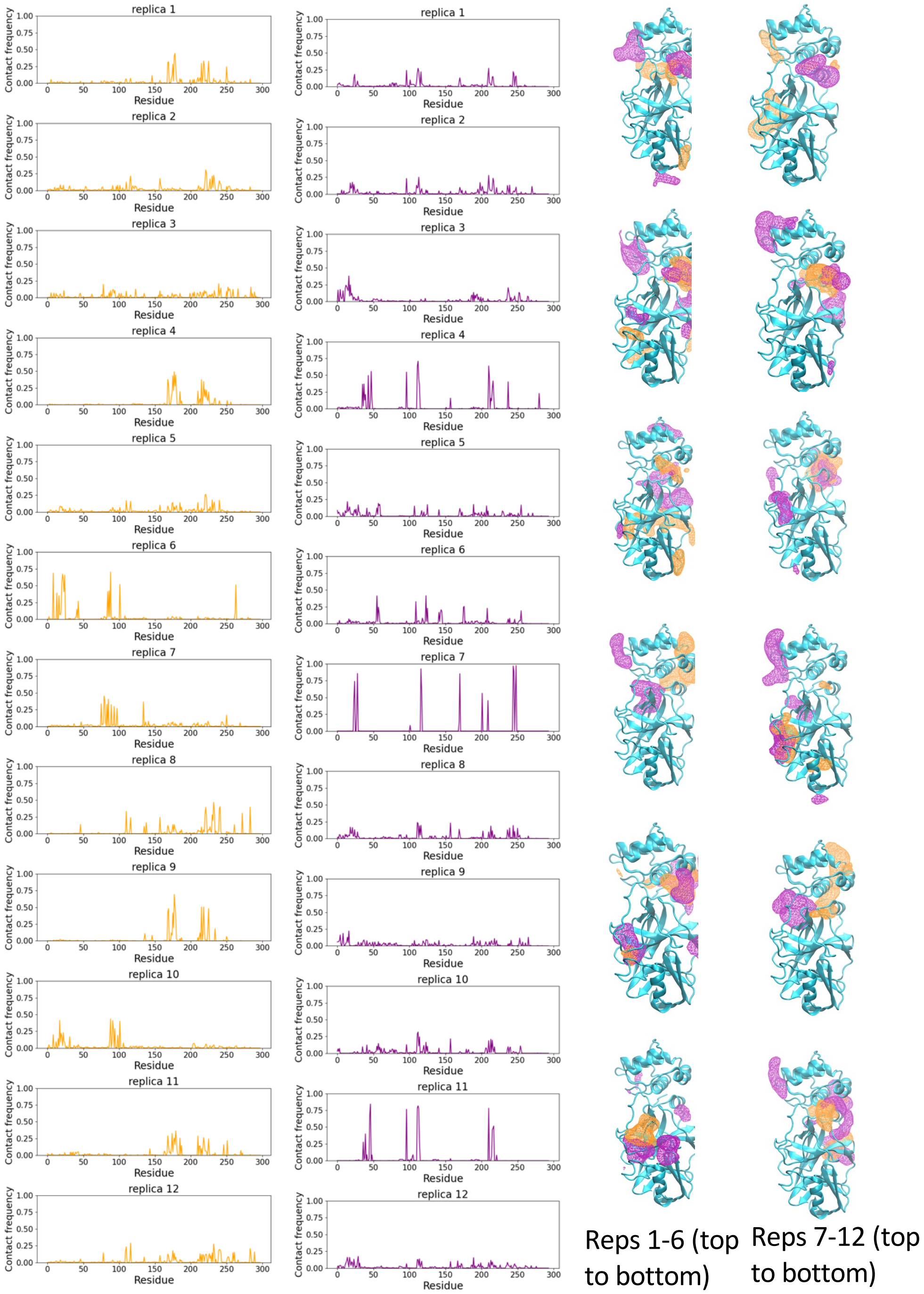

Figure S4: Ligand-protein residue contact frequency distribution plots for each "long" replica are shown along with their respective ligand occupancy maps for the 3CLPro-RQN-LZE system. The ligand-residue contact frequencies correspond to the fraction of frames in which a hydrophobic contact is formed between the ligand and a given protein residue. Occupancy maps of the ligand around the protein represent the isovalue surfaces (wireframe representation) rendered at a fractional occupancy of 0.03 across all frames of the simulation trajectory. In other words, they represent volumes of the simulation box where the ligand is likely to be found with 97% probability, that is in 97% of all trajectory frames.

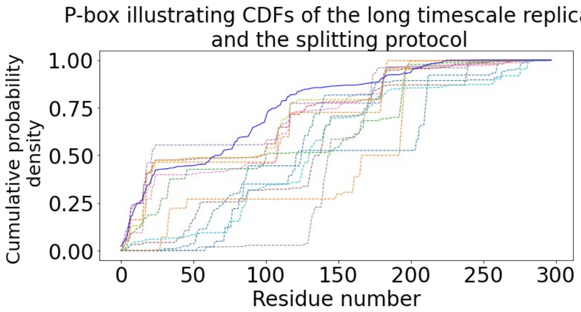

(a) 3CLPro-93J

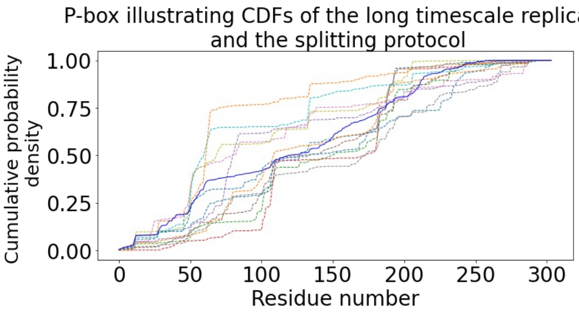

(b) 3CLPro-RQN

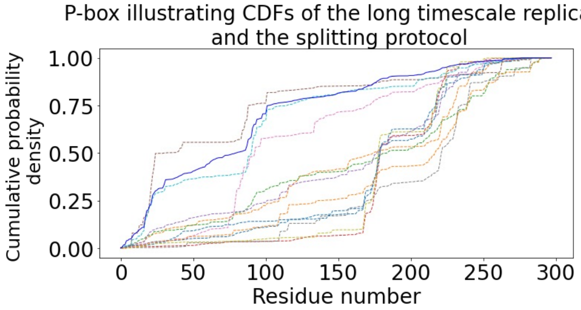

(c) 3CLPro-RQN (with LZE)

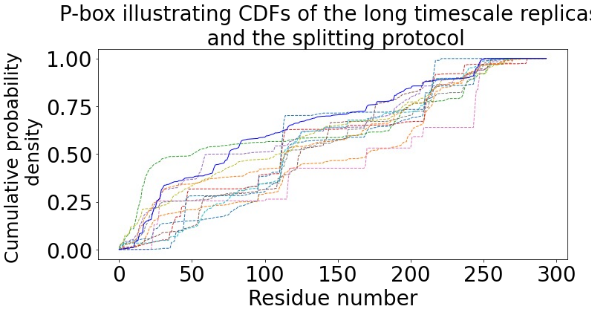

(d) 3CLPro-LZE (with RQN)

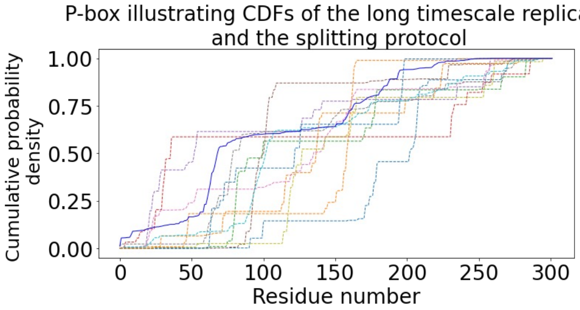

(e) PLPro-GRL

Figure S5: Cumulative density functions (CDFs) of the contact frequency distributions for all "long" replicas (dashed lines) as well as concatenated splitting protocol trajectories (solid line) for all but ADRP systems studied (a-e). The width of the p-box so generated indicates the extent of variability across 'long' replicas compared against the splitting protocol.

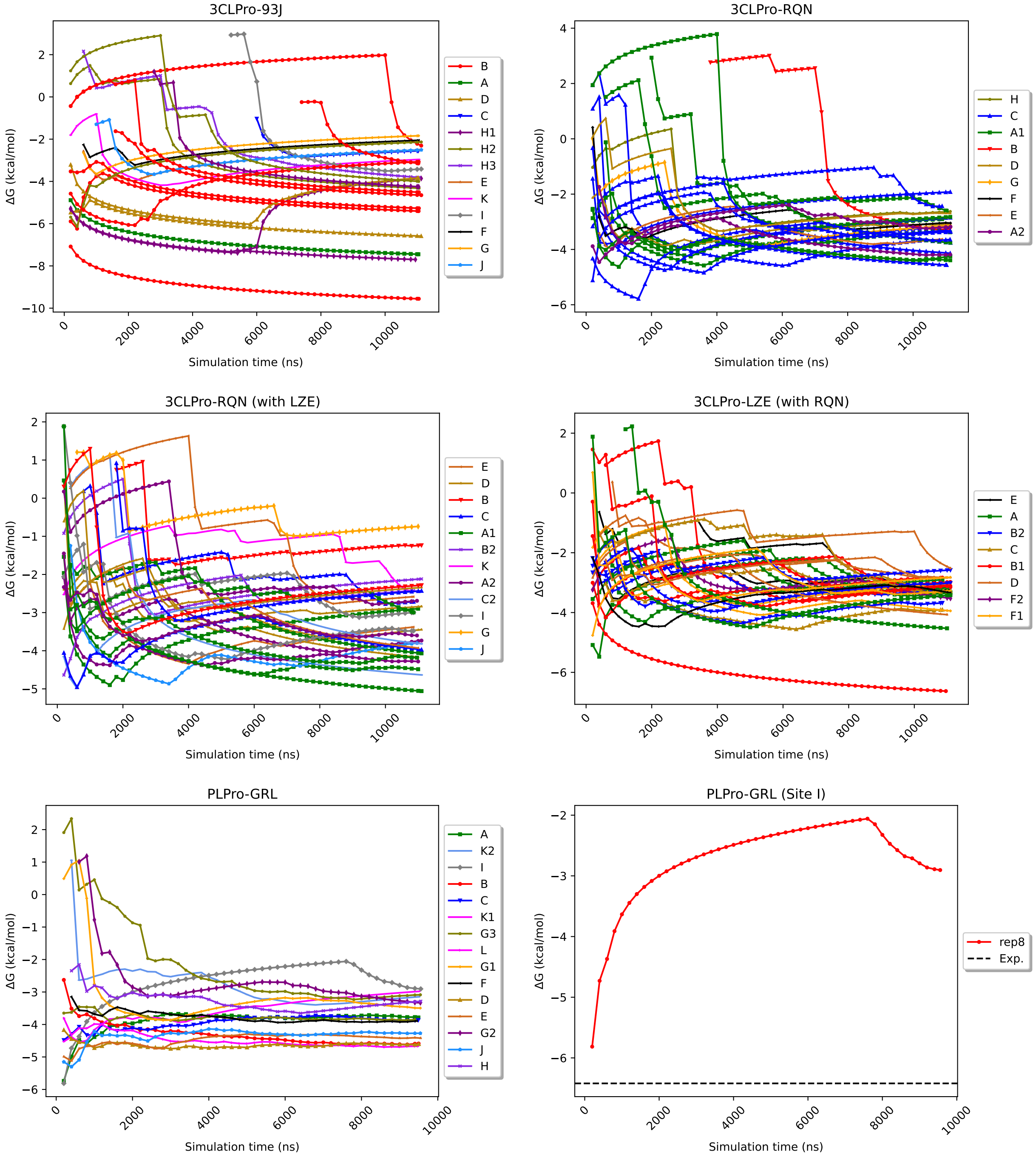

Figure S6: Running averages of ΔG_{direct} for all but ADRP complexes studied. Legends denote replica ID (bottom-right panel) or binding sites (all other panels). The dashed black line denotes the experimental ΔG value.

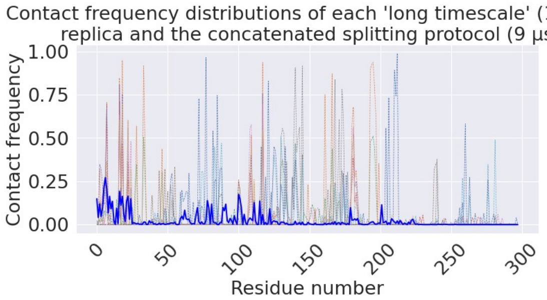

(a) 3CLPro-93J

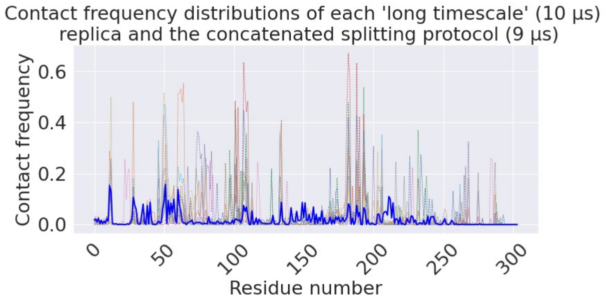

(b) 3CLPro-RQN

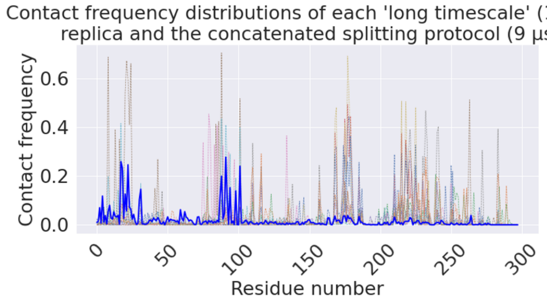

(c) 3CLPro-RQN (with LZE)

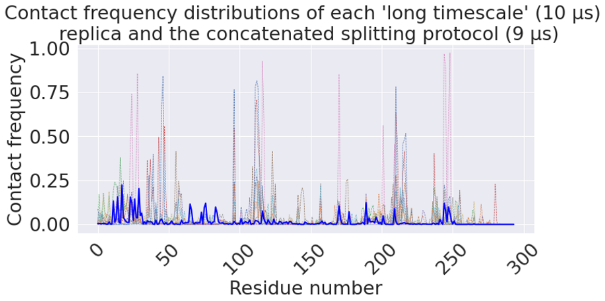

(d) 3CLPro-LZE (with RQN)

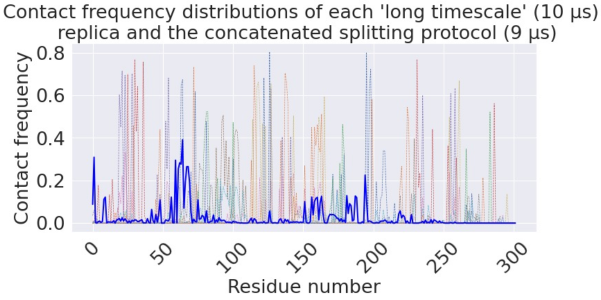

(e) PLPro-GRL

Figure S7: Contact frequency distributions of all "long" (10 μs) replicas (dashed lines) compared to that of the splitting protocol (9 μs) (solid blue line) for all but ADRP systems studied (a-e).

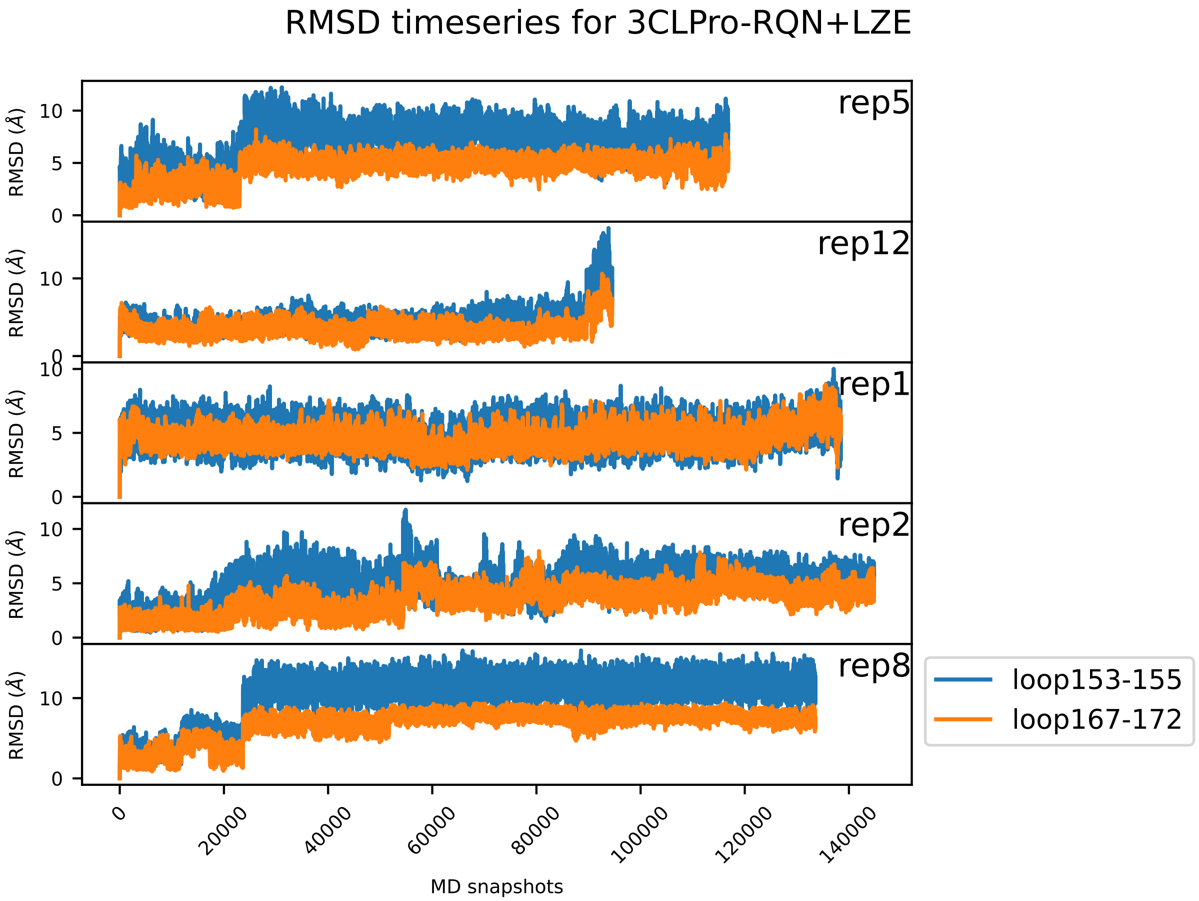

Figure S8: Timeseries of RMSD values for loops 153-155 and 167-172 of 3CLPro-LZE (with RQN) complex for the 5 replicas where LZE binds to site B1 taking only the bound frames.

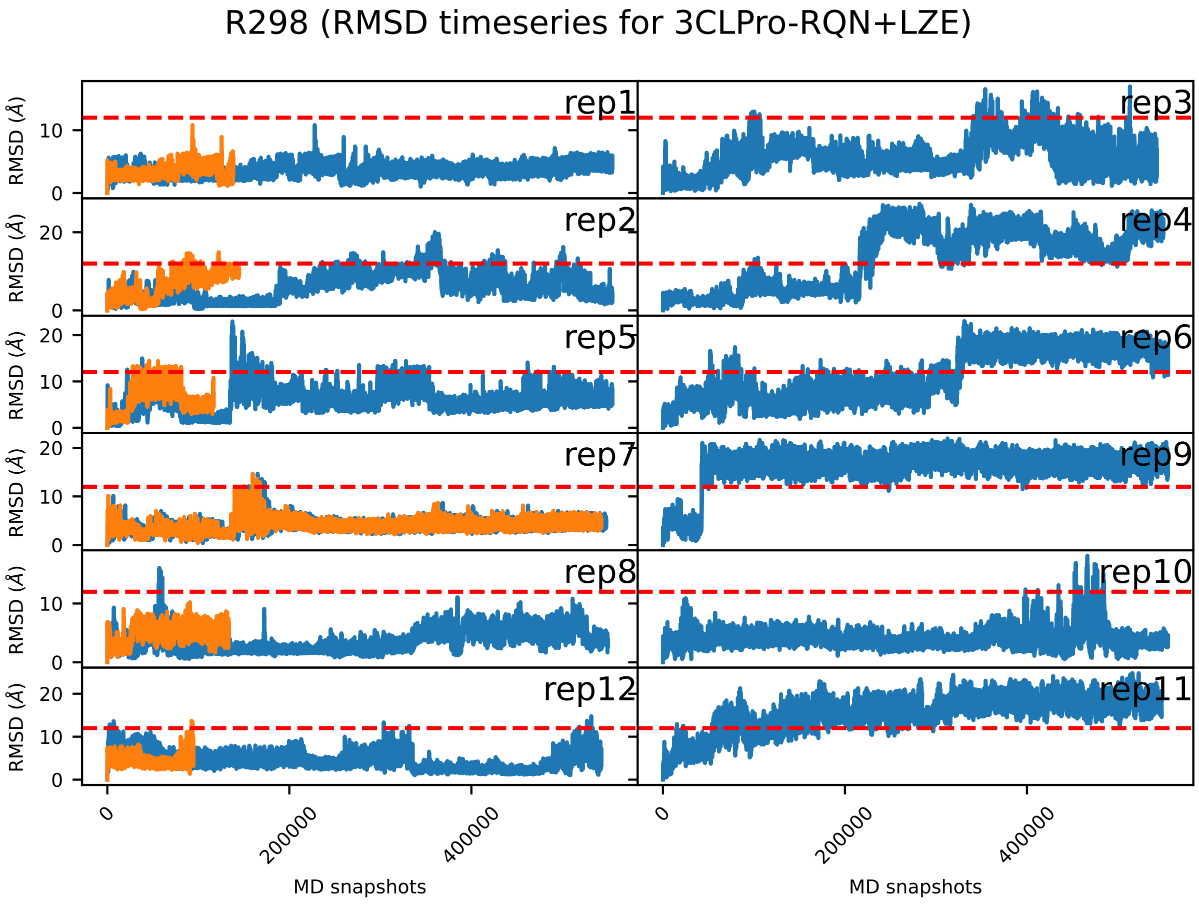

Figure S9: Timeseries of RMSD values for ARG298 residue of 3CLPro-RQN (with LZE) complex for all 12 replicas (shown in blue). The replicas displayed in the left column are the ones where LZE binds at site B1 for which the timeseries of only the bound frames are also included in orange. The dashed red line corresponds to the RMSD value of 12 Å. It is evident that RQN binding triggers large conformational changes corresponding to elevated RMSD values in some replicas. Interestingly, this behaviour is not found in any replica where LZE binds at site B1.

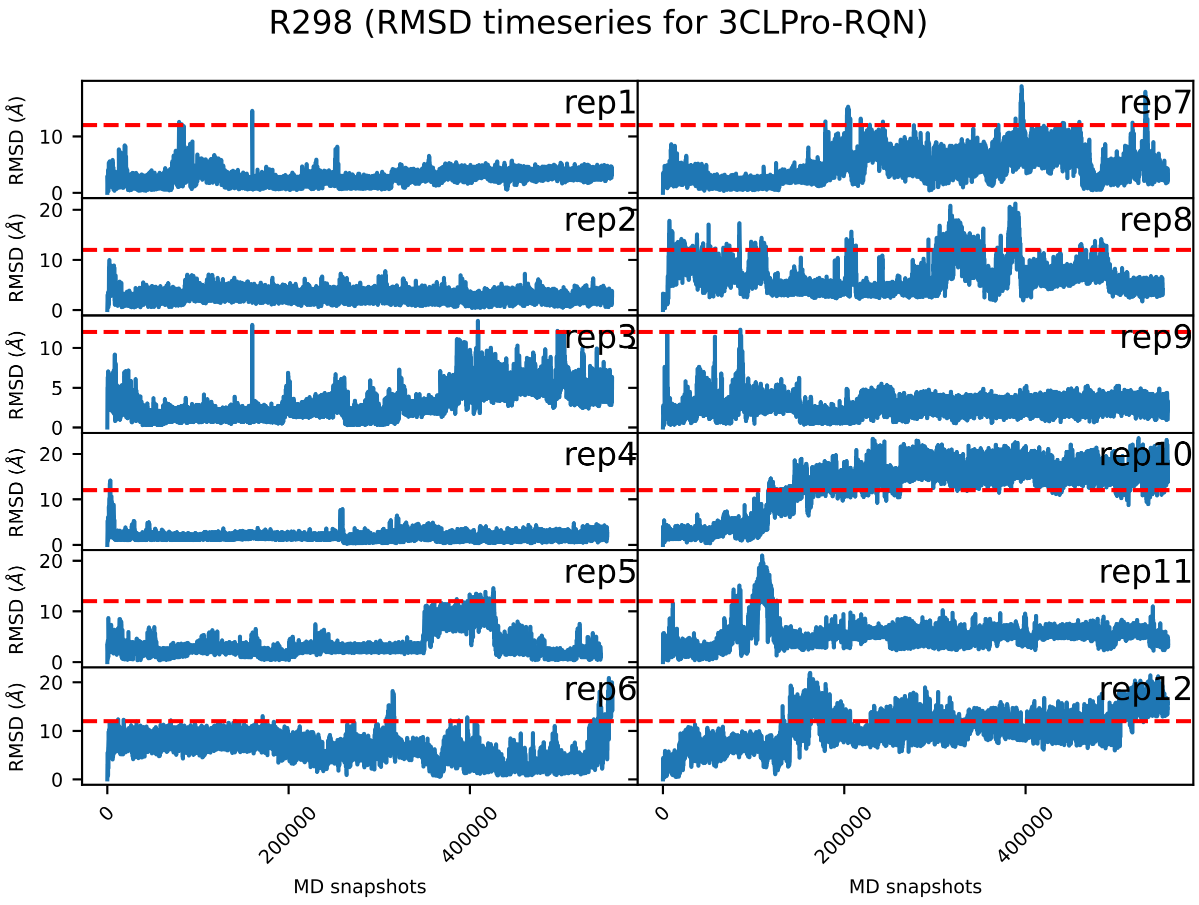

Figure S10: Timeseries of RMSD values for ARG298 residue of 3CLPro-RQN (without LZE) complex for all 12 replicas. The dashed red line corresponds to the RMSD value of 12 Å. It is evident that RQN binding triggers large conformational changes corresponding to elevated RMSD values in some replicas.

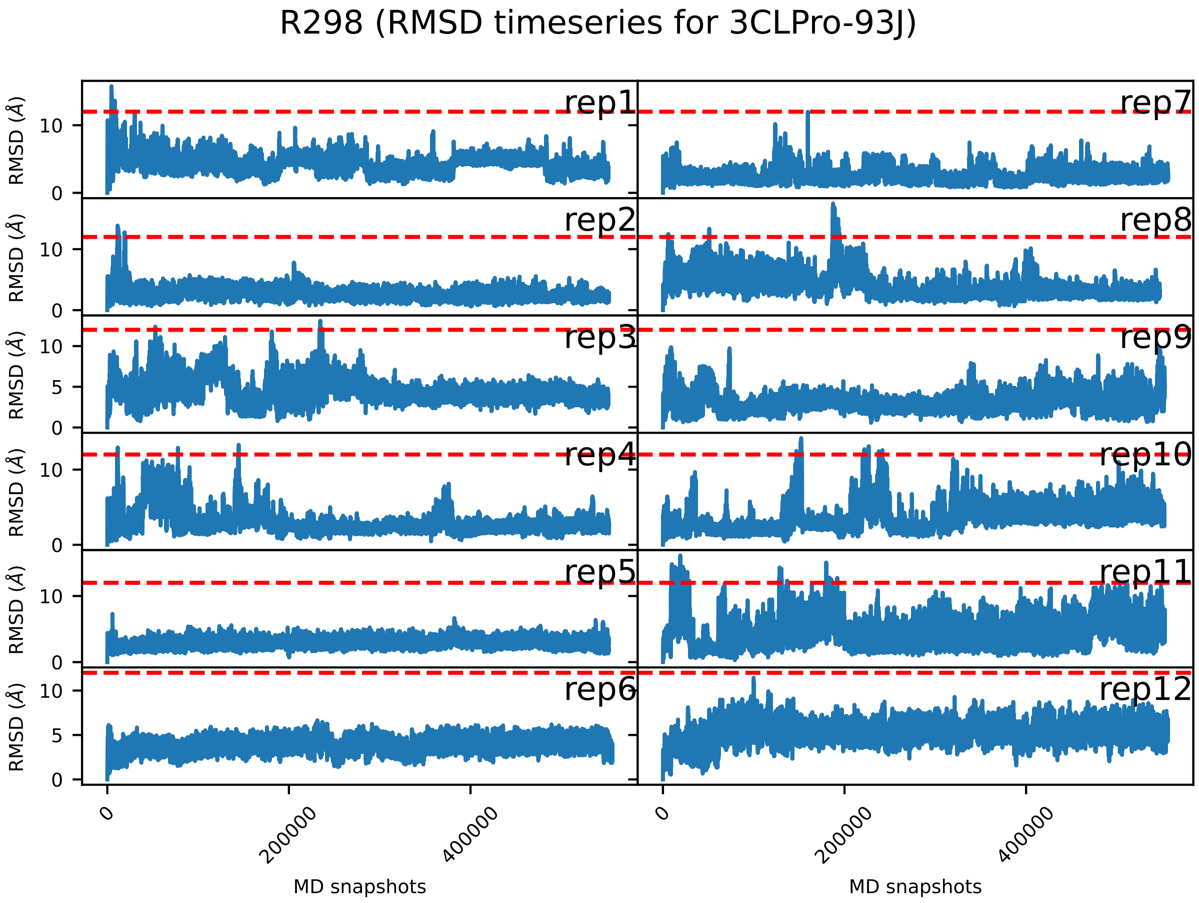

Figure S11: Timeseries of RMSD values for ARG298 residue of 3CLPro-93J complex for all 12 replicas. The dashed red line corresponds to the RMSD value of 12 Å. It is evident that none of the replicas have large RMSD values indicating that the corresponding conformational changes are specific to RQN binding.
